# Pannexin 1 channels regulate leukocyte emigration through the venous endothelium during acute inflammation

**DOI:** 10.1038/ncomms8965

**Published:** 2015-08-05

**Authors:** Alexander W. Lohman, Igor L. Leskov, Joshua T. Butcher, Scott R. Johnstone, Tara A. Stokes, Daniela Begandt, Leon J. DeLalio, Angela K. Best, Silvia Penuela, Norbert Leitinger, Kodi S. Ravichandran, Karen Y. Stokes, Brant E. Isakson

**Affiliations:** 1 Department of Molecular Physiology and Biological Physics, University of Virginia School of Medicine, Charlottesville, Virginia 22908, USA; 2 Robert M. Berne Cardiovascular Research Center, University of Virginia School of Medicine, Charlottesville, Virginia 22908, USA; 3 Department of Molecular and Cellular Physiology, Louisiana State University Health Sciences Center, Shreveport, Los Angeles 71130, USA; 4 British Heart Foundation Cardiovascular Research Centre, College of Medical, Veterinary and Life Sciences, University of Glasgow, Glasgow G12 8TA, UK; 5 Department of Pharmacology, University of Virginia School of Medicine, Charlottesville, Virginia 22908, USA; 6 Department of Anatomy and Cell Biology, University of Western Ontario, London, Ontario, Canada N6A 5C1; 7 Department of Microbiology, Immunology and Cancer Biology, University of Virginia School of Medicine, Charlottesville, Virginia 22908, USA

## Abstract

Inflammatory cell recruitment to local sites of tissue injury and/or infection is controlled by a plethora of signalling processes influencing cell-to-cell interactions between the vascular endothelial cells (ECs) in post-capillary venules and circulating leukocytes. Recently, ATP-sensitive P_2_Y purinergic receptors have emerged as downstream regulators of EC activation in vascular inflammation. However, the mechanism(s) regulating cellular ATP release in this response remains elusive. Here we report that the ATP-release channel Pannexin1 (Panx1) opens downstream of EC activation by TNF-α. This process involves activation of type-1 TNF receptors, recruitment of Src family kinases (SFK) and SFK-dependent phosphorylation of Panx1. Using an inducible, EC-specific Panx1 knockout mouse line, we report a previously unidentified role for Panx1 channels in promoting leukocyte adhesion and emigration through the venous wall during acute systemic inflammation, placing Panx1 channels at the centre of cytokine crosstalk with purinergic signalling in the endothelium.

## Abstract (web summary)

Endothelial cell activation by inflammation requires extracellular ATP release. Here the authors show that TNF-α induces Src-family kinase-dependent ATP release by Pannexin1 channels in endothelial cells, and that Pannexin1 is required for leukocyte adhesion and emigration into the inflamed tissue.

## Graphical abstract

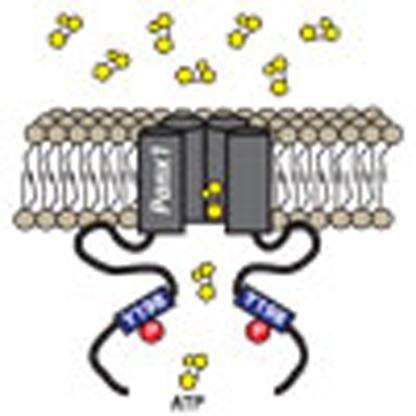

Acute vascular inflammation is a central physiological host defence and repair system that encompasses the innate trafficking and targeting of circulating inflammatory cells (primarily neutrophils) to local sites of tissue injury or infection (see ref. 1). The vascular inflammatory response is essential for the homeostatic balance of cellular turnover and proper clearance of potentially hazardous pathogens and necrotic cell debris that accompanies the resolution of traumas.

As our understanding of the acute inflammatory response has expanded, a prominent role for extracellular signalling by adenosine triphosphate (ATP) and its metabolic breakdown products has emerged. The extracellular accumulation of the purine nucleotides ATP, adenosine diphosphate (ADP) or adenosine can trigger intracellular signalling cascades through the activation of plasma membrane purinergic receptors^2^. Recently, using pharmacological inhibitors and genetically modified mice, Zerr *et al.* identified a pivotal role for vascular purinergic receptor P2Y_1_ in signalling the pro-inflammatory effects of tumour necrosis factor-alpha (TNF-α) and interleukin-1β (IL-1β). When challenged with TNF-α and IL-1β, mice lacking P2Y_1_ receptors displayed a significant reduction in leukocyte recruitment^3^. Furthermore, inhibiting P2Y_1_ function in isolated murine endothelial cells (ECs) prevented the TNF-α-dependent upregulation of adhesion molecules including P-selectin, VCAM1 and ICAM1 (ref. 3). In a separate study, the endothelial P2Y_6_ receptor was shown to control TNF-α-induced inflammatory gene transcription, where pharmacological inhibition of P2Y_6_ receptors potently reduced NFκB activity and downstream transcription of the pro-inflammatory cytokine IL-8 and VCAM1 (ref. 4). Moreover, mice genetically lacking P2Y_6_ had reduced VCAM1 expression and preserved EC barrier integrity when challenged with lipopolysaccharide, a bacterial toxin that increases plasma TNF-α levels^4^. As extracellular ATP concentrations increase, ecto-enzymes at the EC:leukocyte surface actively degrade the purine to ADP, adenosine monophosphate (AMP) and adenosine. The ectonucleoside triphosphate diphosphohydrolase CD39 (ecto-apyrase) degrades ATP and ADP to AMP, while 5′-nucleotidase (CD73) functions to remove the terminal phosphate from AMP yielding adenosine. Consistent with reports implicating purinergic receptors in the vascular inflammatory response, these ecto-enzymes have also been reported to modulate leukocyte recruitment in a number of models of inflammation. For example, mice deficient in CD39 (ecto-apyrase) exhibit enhanced leukocyte targeting to sites of inflammation in the liver and lung^5^,^6^,^7^. In addition, mice lacking CD73 have exacerbated leukocyte–EC interactions during inflammatory stress, and multiple lines of evidence now indicate adenosine as an anti-inflammatory molecule^8^,^9^. On the basis of these observations, purinergic mechanisms play an important role in regulating vascular inflammation and the relative abundance of extracellular ATP and adenosine balances pro- and anti-inflammatory signalling processes; however, the precise mechanism(s) that mediate/regulate ATP release during this physiological response remains unknown.

The pannexin (Panx) family of channel-forming proteins are expressed in the vasculature^10^,^11^,^12^,^13^. Panxs exist in three isoforms (Panx1, Panx2 and Panx3), which are differentially expressed throughout the body. Panx1, the most highly expressed member in the vascular wall, is thought to form hexameric channels permeable to ions and metabolites up to ∼1 kDa in size, including ATP^14^,^15^. To date, the major function of these channels has been ascribed to the release of ATP and as a result, Panx1 channels provide a strong candidate for vascular ATP release during inflammation. Outside of vascular cells, Panx1 channel function has been implicated in several inflammatory processes^16^. In particular, Panx1 channels promote activation of the inflammasome in macrophages, neurons and astrocytes^17^,^18^, regulate chemotaxis of phagocytes during apoptosis^19^, promote T-cell activation^20^, induce neuronal death during enteric colitis^21^ and regulate lung inflammation^22^. Collectively, these studies indicate increased Panx1 channel function during inflammatory stress and provide a framework for understanding the link between cytokine and purinergic signalling pathways in a number of organ systems. The focus of this work is to define the mechanism of ATP release from vascular ECs during inflammatory activation, and address whether Panx1 channels provide the conduit for nucleotide release to promote interactions between circulating inflammatory cells and the endothelium, which has been previously undefined.

Here we show that EC Panx1 channels release ATP in response to activation by the pro-inflammatory cytokine TNF-α, which requires activation of type-1 TNF receptors and downstream signalling through Src family kinases (SFK). The release of ATP promotes upregulation of Panx1 phosphorylation at Y198 and vascular cell adhesion molecule 1 (VCAM1), which regulates leukocyte adhesion and diapedisis through the post-capillary venule wall.

## Results

### TNF-α induces ATP release from venous ECs

**Figure 1:**
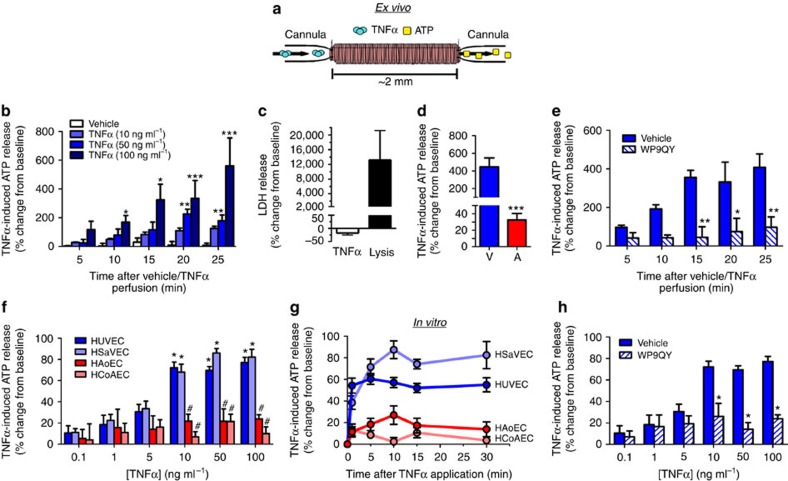
Venous endothelial cells release ATP when activated by TNF-α. (**a**) Schematic of *ex vivo* vascular perfusion assay. (**b**) TNF-α-induced ATP release from isolated murine mesenteric venules. TNF-α promoted a time- and dose-dependent increase in ATP release from the endothelium. *P<0.05, **P<0.01 and ***P<0.001 as compared with vehicle-perfused controls (*n*=4). (**c**) LDH release from isolated venules perfused with TNF-α or lysis buffer. (**d**) ATP release from isolated mesenteric venules (V) and paired arterioles (A) in response to TNF-α (50 ng ml^−1^) perfusion. ***P<0.001 versus venule (*n*=4). (**e**) Time course of ATP release from mesenteric venules following inhibition of TNFR1 with WP9QY (10 μM). *P<0.05 and **P<0.01 versus the corresponding vehicle time point (*n*=4). (**f**) Dose response of primary human venous (HUVEC and HSaVEC) and arterial (HAoEC and HCoAEC) ECs to TNF-α. HUVEC, human umbilical vein endothelial cell; HSaVEC, human saphenous vein endothelial cell; HAoEC, human aortic endothelial cell; HCoAEC, human coronary artery endothelial cell. *P<0.01 compared with unstimulated cells and ^#^*P*<0.005 as compared with venous cells (*n*=5). (**g**) Time course of ATP release from cultured arterial and venous ECs. Cells were stimulated with 10 ng ml^−1^ TNF-α. (**h**) Dose response of HUVEC to TNF-α following inhibition of TNFR1 with WP9QY (10 μM). *P<0.05 as compared with vehicle control (*n*=5). All data are presented as mean±s.e.m. (error bars). Statistical analyses were performed using one-way analysis of variance.

To test whether post-capillary ECs provide a releasable pool of ATP in response to activation by inflammatory mediators, we assessed ATP release in response to activation by the pro-inflammatory cytokine TNF-α. First, we developed an *ex vivo* vascular perfusion assay to selectively deliver TNF-α to the endothelium of intact murine arterioles and venules. Following microdissection of paired second-order mesenteric arterioles and venules, vessels were cannulated on glass micropipettes mounted in a temperature-controlled bath allowing access to the lumen and direct perfusion to a collection reservoir for sample acquisition (Fig. 1a). Perfusion of recombinant murine TNF-α through the lumen of mesenteric venules produced a time- and dose-dependent increase in ATP accumulation in the perfusate as assessed by luciferin:luciferase-based bioluminescence (Fig. 1b). Perfusion of TNF-α did not induce cell death evidenced by a lack of lactate dehydrogenase (LDH) accumulation in perfusates (Fig. 1c). Interestingly, this response was absent in paired mesenteric arterioles (Fig. 1d). The endothelium primarily senses soluble TNF-α via TNF receptor type 1 (TNFR1); therefore, we used a peptide antagonist of this receptor (WP9QY) to examine whether ATP release occurs via a TNFR1-dependent mechanism. Lumenal perfusion of WP9QY prior to EC activation with TNF-α significantly ablated the TNF-α-induced ATP release from intact venous endothelium, indicating a direct involvement of this receptor isoform (Fig. 1e). To further dissect the molecular mechanisms controlling ATP release in this response, we performed *in vitro* ATP release assays on two independent primary venous EC types derived from human umbilical vein (HUVEC) and human saphenous vein (HSaVEC) cells. Following activation with recombinant human TNF-α, we observed a significant increase in ATP accumulation in the supernatant surrounding both HUVEC and HSaVEC monolayers (Fig. 1f,g). Similar to isolated venules, TNF-α stimulation did not activate caspase-dependent cell death pathways in cultured cells, as evidenced by lack of inhibition in ATP release with the pan-caspase inhibitor Q-VD-OPh (Supplementary Fig. 1). Consistent with the intact mesenteric venous endothelium, ATP release from cultured venous ECs increased in a dose- and time-dependent manner with maximal accumulation of extracellular ATP achieved following EC activation with 10 ng ml^−1^ TNF-α for ∼10 min. Similar to *ex vivo* analyses, two primary arterial EC types, human aortic (HAoEC) and human coronary artery (HCoAEC), failed to release ATP upon TNF-α stimulation (Fig. 1f,g). This difference in response was not due to differential expression of TNFR1 in the isolated arteriole:venule pairs or the primary ECs (Supplementary Fig. 2a,b). Moreover, blockade of TNFR1 signalling with WP9QY reduced the ATP release in response to increasing doses of TNF-α on cultured venous ECs, consistent with the *ex vivo* observations (Fig. 1h; Supplementary Fig. 4a). Next, we aimed to examine the ability of another prominent pro-inflammatory cytokine IL-1β to induce ATP from venous ECs. In contrast to TNF-α, IL-1β failed to promote ATP release from HUVEC and HSaVEC monolayers (Supplementary Fig. 3a,b). Co-administration of IL-1β with TNF-α did not significantly alter the degree of ATP release as compared with TNF-α stimulation alone, suggesting a mechanism specific to TNFR1 activation (Supplementary Fig. 3c,d). Taken together, these results provide new evidence for the ability of TNF-α to induce ATP release from venous ECs through a signalling process specific to activation of the membrane receptor TNFR1.

### Panx1 controls ATP release by venous ECs

**Figure 2:**
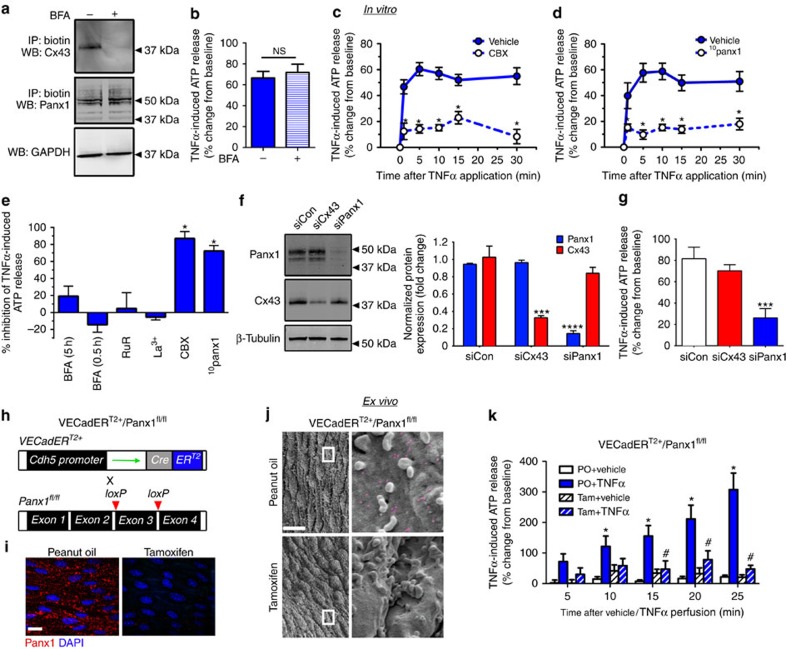
Pannexin 1 channels mediate TNF-α-induced ATP release from venous ECs. (**a**) Representative western blot of HUVEC treated with brefeldin A (BFA; 5 h) and subsequent cell surface biotinylation of membrane proteins. Plasma membrane localization of Panx1 and Cx43 were assessed using isoform-specific antibodies to each protein. (**b**) ATP release from BFA-treated HUVEC in response to TNF-α (10 ng ml^−1^) treatment for 30 min. (**c**,**d**) Time course of ATP release from HUVEC following inhibition of Panx1 channels with carbenoxolone (CBX: 50 μM) (**c**) and the Panx1 blocking peptide ^10^panx1 (200 μM) (**d**). *P<0.05 as compared with vehicle control (*n*=5). (**e**) Summary data of pharmacological inhibitors assessed for inhibition of TNF-α-induced ATP release from HUVEC. BFA (30 min): inhibition of vesicular release, Ruthenium red (RuR): antagonist of CALHM1 channels. Lanthanum (La^3+^): Cx hemichannel antagonist. *P<0.05 as compared with BFA, RuR and La^3+^ (*n*=5). (**f**) Representative western blots of siRNA knockdown of Panx1 and Cx43 in HUVEC. ***P<0.005 and ****P<0.001 versus control (*n*=3). (**g**) ATP release from Panx1 and Cx43 siRNA-treated HUVEC from **f** in response to TNF-α (10 ng ml^−1^). ***P<0.005 versus control (*n*=3). (**h**) Schematic representing the generation of an inducible, EC-specific Panx1 knockout mouse (*VECadER*^*T2+*^*/Panx1*^*fl/fl*^). (**i**) *En face* immunofluorescence micrographs of Panx1 (red) expression of endothelium from *VECadER*^*T2+*^*/Panx1*^*fl/fl*^ mice injected with tamoxifen (Tam) or its vehicle peanut oil (PO) for 10 consecutive days. Nuclei are stained with 4,6-diamidino-2-phenylindole (DAPI) (blue). Scale bar, 10 μm. (**j**) Immuno-scanning electron micrographs (iSEM) of isolated mesenteric venules from *VECadER*^*T2+*^*/Panx1*^*fl/fl*^ mice. Veins were immunolabelled for Panx1 (pseudo-coloured magenta) using an antibody against the extracellular region of the channel. Right panels are zoomed images of the left panels. Scale bar, 10 μm; enlarged boxes are 5 × 5 μm. (**k**) TNF-α-induced ATP release from isolated mesenteric venules from *VECadER*^*T2+*^*/Panx1*^*fl/fl*^ mice injected with Tam or PO for 10 days. Venules were perfused with 50 ng ml^−1^ TNF-α or vehicle. *P<0.05 as compared with vehicle-perfused controls and ^#^*P*<0.05 as compared with PO+TNF-α (*n*=4). All data are presented as mean±s.e.m. (error bars). Statistical analyses were performed using one-way analysis of variance. NS, not significant.

We next sought to identify the specific pathway regulating ATP release activated by TNF-α. Multiple regulated ATP release pathways have been reported in the vasculature, including prominent roles for vesicular and channel-dependent ATP release primarily involving connexins (Cx) hemichannels and Panx1 channels^23^ (Panx1 is the predominant isoform expressed in the systemic vasculature^10^; Supplementary Fig. 2c). These two protein families share a similar membrane topology, and distinguishing connexin hemichannels from pannexin channels in cellular ATP release has often proved cumbersome due to overlapping expression profiles in a number of cell types and the lack of specific pharmacological inhibitors^24^. Therefore, we took a multifaceted approach to dissect the TNF-α-mediated ATP release pathway. Initially, to discriminate between the involvement of connexin hemichannels and Panx1 channels in this process, we used a biochemical method to selectively deplete connexins from the EC plasma membrane without affecting the localization of Panx1. Taking into account the relative half-life of each channel at the cell surface (Cx hemichannels: 1–5 h (ref. 25), Panx1 channels: >6 h (ref. 26)), treatment of HUVEC and HSaVEC with the vesicular exocytosis inhibitor brefeldin A (BFA; 5 μg ml^−1^) prevented trafficking of newly synthesized Cx hemichannels and Panx1 channels to the cell surface while preserving internalization of older channels. Due to the relatively short half-life of Cx43 hemichannels (the major Cx isoform implicated in ATP release), 5 h treatment with BFA decreased the expression of Cx43 at the EC surface without significantly altering the expression of Panx1 in both HUVEC (Fig. 2a) and HSaVEC (Supplementary Fig. 4b). Importantly, under these experimental conditions, the ability of TNF-α to induce ATP release was unaffected, suggesting that Panx1 channels may be more likely to mediate ATP release in response to the inflammatory cytokine (Fig. 2b; Supplementary Fig. 4c).

To more directly interrogate the contribution of Panx1 channels to ATP release from venous ECs, we used two independent Panx1 pharmacological antagonists to block channel function, carbenoxolone (CBX) and the Panx1 inhibitory peptide ^10^panx1. In HUVEC, Panx1 channel blockade with CBX (50 μM) or ^10^panx1 (200 μM) significantly reduced TNF-α-mediated ATP release by 86.4 and 70.9%, respectively (Fig. 2c,d). Similar inhibition was observed in HSaVECs (CBX: 69.7%, ^10^panx1: 75.2%; Supplementary Fig. 4d). Furthermore, blockade of Cx hemichannels with lanthanum (La^3+^;100 μM) failed to reduce ATP release (Fig. 2e). Additional pharmacological interrogation ruled out the involvement of vesicular mechanisms (blocked with BFA) and calcium homeostasis modulator 1 (CALHM1) channels (inhibited with ruthenium red^27^), which have recently been identified as potential ATP release channels (Fig. 2e; Supplementary Fig. 4d). Moreover, employing RNA interference, selective knockdown of Panx1 in HUVEC and HSaVEC with short interfering RNA (siRNA) duplexes targeting the *PANX1* gene affirmed a central role for these channels in TNF-α-induced ATP release, with no observed inhibition upon Cx hemichannel depletion with Cx43 siRNA. Knockdown efficiency was ∼65 and ∼75% in HUVEC (Fig. 2f) and HSaVEC (Supplementary Fig. 4e), respectively. In both cell types, Panx1 knockdown significantly attenuated TNF-α-induced ATP release (Fig. 2g; Supplementary Fig. 4f). Finally, we assessed dye uptake by venous ECs as another output for Panx1 activity. Incubation of TNF-α-stimulated HUVEC with YO-PRO-1 produced a significant increase in intracellular dye accumulation in a dose-dependent manner (Supplementary Fig. 5a,b). This effect was significantly attenuated under conditions of Panx1 blockade with ^10^panx1 (Supplementary Fig. 5c). Moreover, addition of YO-PRO-1 to HUVEC monolayers 10 min or later following TNF-α stimulation resulted in a significant decrease in dye uptake as compared with conditions where YO-PRO-1 was present at the onset of TNF-α stimulation (Supplementary Fig. 5d). These data, along with the rapid and transient ATP release observed, suggest that EC Panx1 activation is transient in nature with the channels closing within 10 min of becoming activated by TNF-α-dependent signalling mechanisms.

To directly investigate the contribution of endothelial Panx1 channels to TNF-α-induced ATP release in the intact venous circulation, we engineered mice that specifically lack Panx1 expression in ECs. This was accomplished by crossing mice carrying *loxP* sites flanking exon 3 of the murine *Panx1* gene (*Panx1*^*fl/fl*^)^28^ with transgenic mice carrying a tamoxifen-sensitive Cre recombinase driven by the vascular EC cadherin promoter (*VECadER*^*T2+*^) (Fig. 2h). Because this Cre is basally inactive until tamoxifen treatment, this allowed the mice to develop normally and provided the ability to induce deletion of Panx1 specifically in ECs at the time of our choosing. Following 10 days of tamoxifen administration, *VECadER*^*T2+*^*/Panx1*^*fl/fl*^ mice displayed a substantial reduction in Panx1 expression in the endothelium, as assessed by immunofluorescence microscopy and immune transmission electron microscopy (iTEM) (Fig. 2i,j). Luminal perfusion of TNF-α in isolated mesenteric venules from these mice displayed a marked inhibition of ATP release compared with littermate controls (that is, injected with the vehicle peanut oil (PO) only) (Fig. 2k). Taken together, these data suggest a direct role for Panx1 channels in releasing ATP from venous ECs in response to TNF-α.

### SFK phosphorylate Panx1 in ECs

**Figure 3:**
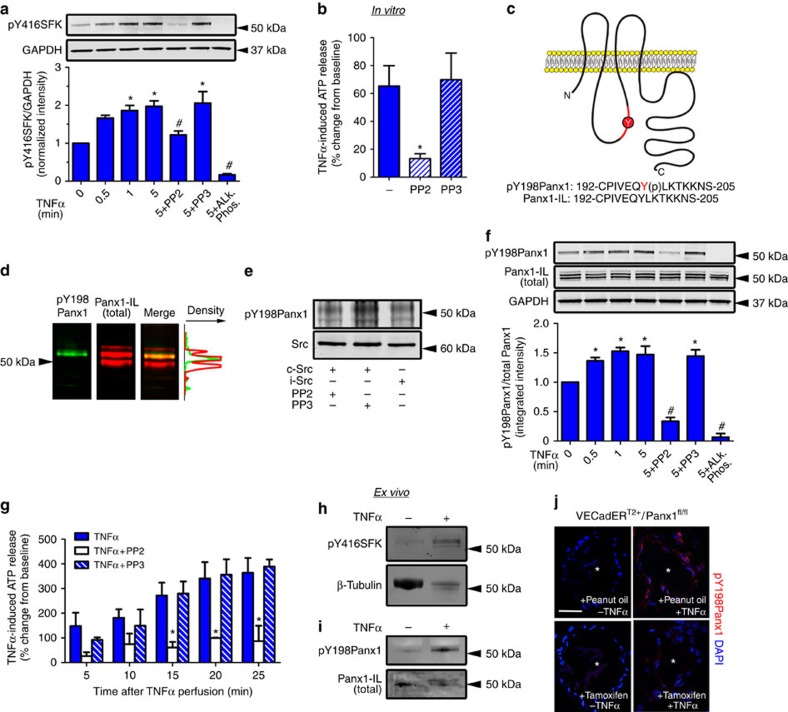
TNF-α induces Src family kinase-dependent activation of EC Panx1 channels. (**a**) Western blot analysis of SFK activation in HUVEC in response to TNF-α stimulation (10 ng ml^−1^). A phospho-specific antibody against Y416 in SFKs (pY416SFK) was used as an indicator of SFK activation. SFK activation was blocked with the pharmacological antagonist PP2 (10 μM) but not by its inactive analogue PP3 (10 μM). Antibody specificity for the phosphorylated form of the kinases was confirmed by dephosphorylating proteins in cell lysates with alkaline phosphatase. *P<0.05 as compared with unstimulated control (lane 1) and ^#^*P*<0.001 as compared with lane 1 (*n*=3). (**b**) TNF-α-induced ATP release from HUVEC following SFK inhibition with PP2. *P<0.05 versus control and PP3 treatments (*n*=5). (**c**) Topological schematic of Panx1 highlighting an epitope in the intracellular loop which contains tyrosine 198. This epitope was used to develop antibodies specific to the phosphorylated (pY198Panx1) and non-phosphorylated (Panx1-IL) forms of the protein. (**d**) Overlay of pY198Panx1 signal and Panx1-IL (total) signal as assessed by western blotting with LiCOR IRDye secondary antibodies. pY198Panx1 detects a single species at ∼55 kDa. (**e**) Western blot analysis of pY198Panx1 in HUVEC transfected with plasmids encoding c-Src and/or inhibitor of Src (i-Src). (**f**) Western blot analysis of Panx1 phosphorylation at Y198 in HUVECs stimulated with TNF-α (10 ng ml^−1^). Phospho-signal was normalized to total Panx1 expression using the Panx1-IL Ab. *P<0.05 compared with vehicle control (lane 1) and ^#^*P*<0.01 compared with 5-min TNF-α stimulation (lane 4) (*n*=3). (**g**) TNF-α-induced ATP release from mesenteric venules treated with PP2 (10 μM) or PP3 (10 μM). *P<0.05 as compared with vehicle control (*n*=5). (**h**,**i**) Western blot analysis of TNF-α-induced SFK activation (**h**) and pY198Panx1 phosphorylation (**i**) in isolated mesenteric venules perfused with TNF-α (50 ng ml^−1^) for 30 min. (**j**) Immunofluorescence micrographs of pY198Panx1 in isolated mesenteric venule cross-sections. Venules were isolated from mice expressing endogenous Panx1 in the vascular wall (*VECadER*^*T2+*^*/Panx1*^*fl/fl*^+peanut oil) or mice with specific EC Panx1 deletion (*VECadER*^*T2+*^*/Panx1*^*fl/fl*^+tamoxifen) and stimulated with TNF-α. Asterisks indicate the vessel lumen and nuclei are stained with 4,6-diamidino-2-phenylindole (DAPI) (blue). Scale bar, 30 μm. All data are presented as mean±s.e.m. (error bars). Statistical analyses were performed using one-way analysis of variance.

Next, we aimed to elucidate the mechanism by which activation of TNFR1 in venous ECs could translate to Panx1 channel opening. TNFR1 activation has been reported to induce the activity of a number of intracellular kinases, including the Src family tyrosine kinases (SFK)^29^,^30^. In addition, endothelial and inflammatory cell SFKs are involved in increasing EC barrier permeability and increasing the recruitment, adhesion and transmigration of circulating neutrophils, monocytes and macrophages to inflamed tissues^31^. Recent analysis of Panx1 activity in hippocampal neurons revealed the involvement of SFKs in NMDA-mediated Panx1 activation^32^. Therefore, we assessed the potential contribution of SFKs in TNF-α-induced Panx1 activation and ATP release from venous ECs. SFK activation can be assessed by autophosphorylation of a conserved tyrosine residue (Y416), which stabilizes a substrate-permissive, active-site conformation in the kinases^33^. Using a phospho-specific Y416SFK antibody, we detected a significant increase in Y416 phosphorylation in both HUVECs (Fig. 3a) and HSaVECs (Supplementary Fig. 4g) following acute (5 min) exposure to recombinant human TNF-α *in vitro*, consistent with the rapid induction of ATP release from these cultured primary cells. This effect was specific to SFK activation as treatment of both venous cell types with the SFK inhibitor PP2 reduced Y416 phosphorylation to baseline levels, while PP3 (the inactive analogue of PP2) had no effect. Functionally, pharmacological inhibition of SFKs with PP2 significantly blunted TNF-α-induced ATP release from both HUVEC (Fig. 3b) and HSaVEC (Supplementary Fig. 4h), while PP3 showed no significant effect. Importantly, examination of SFK activation downstream of TNF-α signalling in isolated mesenteric venules revealed a conserved role for SFKs in promoting ATP release from intact venous endothelium, with PP2 blunting ATP release (Fig. 3g) and TNF-α increasing SFK activation (Fig. 3h).

We next sought to determine whether Panx1 channels are targets for phosphorylation downstream of SFK activation. Recently, our laboratory defined a region of the Panx1 intracellular loop that is important for receptor-mediated channel activation in vascular smooth muscle cells. This intracellular loop region (amino acids 198–200) contains a highly conserved tyrosine residue (Y198), which may constitute a putative SFK phosphorylation site^34^. From these observations, we developed a new phospho-specific Panx1 antibody against Y198 (pY198Panx1) and a control antibody against the same non-phosphorylated epitope (Panx1-IL) (Fig. 3c). The pY198Panx1 antibody detects a singular Panx1 species of ∼55 kDa by western blotting (Fig. 3d). Overexpression of c-Src in HUVECs significantly increased phosphorylation of Panx1 at Y198 as compared with cells expressing an inhibitor of Src (i-Src) (Fig. 3e). In addition, this response could be blocked by inhibition of Src with PP2.

Using this newly developed tool, we addressed Panx1 phosphorylation downstream of TNF-α signalling. Panx1 was phosphorylated downstream of TNF-α-induced SFK activation in ECs. Specifically, treatment of HUVEC and HSaVECs with TNF-α increased phosphorylation at Y198, which could be blocked by pretreatment with the SFK inhibitor PP2 (Fig. 3f; Supplementary Fig. 4i). To directly assess the specificity of this antibody against the phosphorylated form of the channel, we performed dephosphorylation reactions with alkaline phosphatase, which depleted the signal by western blotting. In our *ex vivo* vascular preparations, stimulation of primary ECs with TNF-α also promoted phosphorylation of Panx1 (Fig. 3i). In these studies, isolated mesenteric venules containing both ECs and vascular smooth muscle cells were lysed to produce a heterogeneous sample. To more accurately discriminate between EC and vascular smooth muscle cell (VSMC) Panx1 pools, we performed immunofluorescence microscopy on cross-sections of mesenteric venules using our pY198Panx1 antibody. Mice expressing endogenous levels of Panx1 in the vascular wall (*VECadER*^*T2+*^*/Panx1*^*fl/fl*^+PO) showed a significant increase in Panx1 phosphorylation following stimulation with TNF-α (Fig. 3j). Administration of tamoxifen to a subset of mice abolished this signal, supporting the specificity of pY198Panx1 for Panx1. These observations provide new evidence for phosphorylation of Panx1 in the venous endothelium in the activation of these channels during TNF-α signalling.

### Panx1 activation promotes leukocyte adhesion and emigration

**Figure 4:**
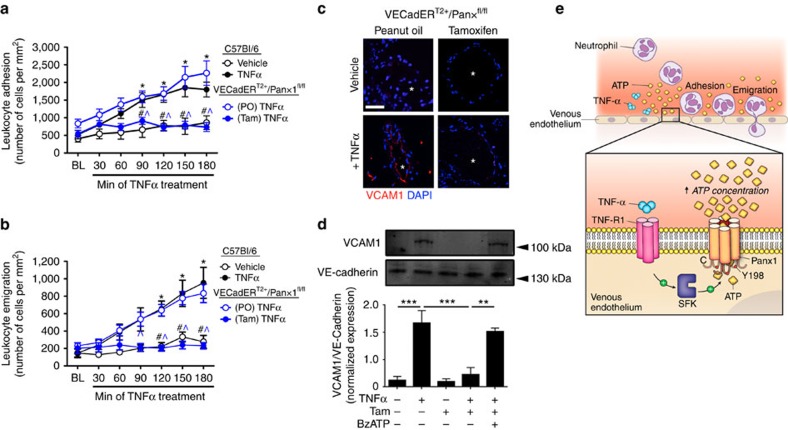
EC Panx1 channels promote leukocyte adhesion and emigration. (**a**,**b**) Quantification of endogenous leukocyte adhesion (**a**) and emigration (**b**) in wild-type (WT) C57Bl/6J mice and mice lacking Panx1 specifically in the endothelium (*VECadER*^*T2+*^*/Panx1*^*fl/fl*^) in the acute inflammatory response. Acute inflammation was induced by topically applying recombinant murine TNF-α to exteriorized cremaster muscles in anaesthetized mice. *P<0.01 as compared with baseline, ^#^*P*<0.005 as compared with C57Bl/6J mice treated with TNF-α and ^ˆ^*P*<0.01 as compared with PO-injected *VECadER*^*T2+*^*/Panx1*^*fl/fl*^ mice treated with TNF-α by two-way analysis of variance (ANOVA) (*n*=5–6 mice per group). (**c**) Immunofluorescence micrographs for VCAM1 expression in isolated mesenteric venules from *VECadER*^*T2+*^*/Panx1*^*fl/fl*^ mice (PO or Tam injected) treated with vehicle or TNF-α (50 ng ml^−1^) for 2 h. * indicates the vessel lumen and nuclei are stained with 4,6-diamidino-2-phenylindole (DAPI) (blue). Scale bar, 30 μm. (**d**) Western blot analysis of VCAM1 expression in isolated mesenteric venules from *VECadER*^*T2+*^*/Panx1*^*fl/fl*^ mice following TNF-α (50 ng ml^−1^) treatment. Exogenous BzATP (10 μM) was applied to assess the potential to rescue VCAM1 upregulation. **P<0.01 and ***P<0.005 by one-way ANOVA (*n*=3). (**e**) Mechanism of TNF-α-induced ATP release from venous ECs in the acute inflammatory response. All data are presented as mean±s.e.m. (error bars).

We next tested whether the EC ATP release mediated via this TNF-α–Panx1 axis may induce leukocyte recruitment to localized inflammatory foci. We initially used an *in vitro* leukocyte adhesion assay. In this assay system, activation of HUVEC monolayers with TNF-α for 30 min increased THP-1 monocyte adhesion by approximately fourfold (Supplementary Fig. 6a,b). This effect was significantly reduced by blockade of TNFR1 (WP9QY), Panx1 channels (CBX and ^10^panx1) and degradation of extracellular ATP (Apyrase). These *in vitro* results suggest a potentiating role for Panx1-dependent ATP release in promoting inflammatory cell interactions with venous ECs. To more directly determine the *in vivo* contribution of Panx1 to acute vascular inflammation, we used intravital microscopy of the exteriorized mouse cremaster muscle. Topical application of TNF-α to the cremaster circulation in C57Bl/6J mice promoted a significant increase in leukocyte interactions with the post-capillary venular endothelium (Fig. 4a,b). Specifically, leukocyte adhesion increased ∼2.5-fold and emigration increased by ∼3.5-fold after 120 min of TNF-α stimulation. Genetic deletion of Panx1 specifically in the endothelium (*VECadER*^*T2+*^*/Panx1*^*fl/fl*^ mice, tamoxifen injected) potently ablated leukocyte adhesion and emigration compared with vehicle (PO only)-injected littermates and wild-type C57Bl/6J control animals. Importantly, the leukocyte rolling *per se* was unaffected after endothelial deletion of Panx1, suggesting that the effects of Panx1 activity are required downstream of the initial rolling stage (Supplementary Fig. 7a). In addition, venule diameter and wall shear rate did not differ between control and Panx1-deleted animals (Supplementary Fig. 7a). In addition, the potent inhibition of adhesion and emigration was not due to off-target effects of tamoxifen administration *per se*, as tamoxifen administration to control C57Bl/6J mice had no effect on these responses (Supplementary Fig. 7b,c).

### Panx1-mediated ATP release upregulates VCAM1

On the basis of the observation that Panx1 activation primarily regulates adhesion and transmigration, we sought to examine the effect of EC Panx1 deletion on the upregulation of VCAM1. VCAM1 is substantially upregulated during EC activation by cytokines and functions to bind its complementary ligand α_4_β_1_ integrin (VLA-4) expressed on circulating leukocytes. Binding of VCAM1 to VLA-4 elicits firm adhesion of circulating cells to the endothelium, allowing downstream emigration into the inflamed tissue. Recently, several studies have emerged implicating ATP and purinergic receptor stimulation in VCAM1 upregulation^35^,^36^,^37^. We performed immunofluorescence microscopy on TNF-α-stimulated mesenteric venules isolated from our EC-specific Panx1 knockout (KO) mice to initially assess the effect on VCAM1 expression. Activation of venules from mice injected with PO increased total VCAM1 expression in the endothelium (Fig. 4c). Conversely, deletion of Panx1 by tamoxifen administration blunted this cytokine-induced VCAM1 upregulation, suggesting a functional role for Panx1 activation in promoting increased expression of the adhesion molecule (Fig. 4c). Furthermore, this effect was due to the release of ATP via Panx1, since VCAM1 upregulation could be rescued in Panx1 KO mice by stimulating with exogenous ATP (Fig. 4d).

Collectively, these new data identify a previously unrecognized signalling pathway implicating Panx1 as a positive regulator of inflammation in the venous endothelium. Specifically, we show that following activation of TNFR1 on venous ECs, SFKs become activated and signal the activation of Panx1 channels. Opening of the Panx1 pore causes ATP liberation from the cells along a concentration gradient where it signals extracellularly to promote leukocyte adhesion to the vascular endothelium and subsequent emigration into the inflamed tissue (Fig. 4e). Physiologically, Panx1 channels represent a potential new target for regulating the purinergic input into inflammatory signalling through TNF-α in the vasculature, and this may prove useful for future pharmacological intervention to regulate inflammatory disorders.

## Discussion

In the cardiovascular system, ATP and its metabolites function extracellularly to regulate the vascular inflammatory response, affecting major aspects of inflammatory signalling in the endothelium including the presentation of adhesion molecules and the integrity of the EC barrier function. While multiple lines of evidence now support a pro-inflammatory role for ATP, the source and mechanism promoting the cellular release of this purine nucleotide is not well defined. Here, using multiple *in vitro*, *ex vivo* and *in vivo* models we provide new evidence identifying Pannexin 1 channels as major conduits for ATP release from the venous endothelium during acute inflammatory stress, with channel activation promoting leukocyte adhesion and emigration across the vessel wall.

TNF-α and IL-1β are the major cytokines that initially regulate vascular cell phenotype during acute systemic inflammation. Increased extracellular concentrations of these signalling molecules is readily observed in a number of inflammatory states, and recent evidence has emerged linking cytokine signalling by TNF-α to purinergic pathways in the vasculature^3^,^4^. Our analysis of cytokine-induced ATP release revealed a selective mechanism by which TNF-α, but not IL-1β, potentiates cellular ATP release from venous ECs. By these means, EC activation by TNF-α and subsequent priming for interactions with circulating inflammatory cells may be favoured by a purinergic amplification step. This is particularly intriguing when considering the dichotomous relationship between cell survival and cell death with respect to TNF-α signalling. This cytokine at low/acute concentrations favours cell survival pathways and homeostatic maintenance at the level of inflammatory cell recruitment through complex I signalling. As such, a purinergic amplification step may promote non-deletarious signalling in the absence of chronic TNF-α exposure. In this respect, acute IL-1β signalling may not require this type of amplification, which is evidenced by the observed lack of ATP release from ECs exposed to this cytokine and lack of a synergistic effect when IL-1β and TNF-α are applied concurrently. This evidence may shed new light on the complexity of inflammatory signalling in the venous circulation prompting further analysis of the interplay between the multiple signalling processes controlling EC activation in the whole animal during inflammation. Our analysis employed a range of TNF-α concentrations (0.1–100 ng ml^−1^) to evaluate the effect on EC ATP release with a significant response observed to doses at 10 ng ml^−1^ and higher. Depending on the inflammatory model tested, measurements of endogenous circulating levels of TNF-α in both humans and animal models have shown considerable variability; however, these values in general fall in the pg ml^−1^–ng-ml^−1^ range. A number of factors likely contribute to this variation including the absolute systemic blood volume, activity of TNF-α-processing enzymes and presence of chelators including soluble TNF receptors. As such, the absolute concentration of TNF-α in the microenvironment near the EC surface is likely underestimated by conventional measurements from serum samples prompting a need for more sensitive techniques for quantifying cytokine concentrations in these local environments.

Our study revealed a venous selectivity to TNF-α-induced ATP release, where ECs of venous origin release ATP following activation by TNF-α with negligible responses in the arterial endothelium. In fact, it has long been observed that inflammatory cell homing to localized tissues occurs almost exclusively in the post-capillary venous circulation, while these interactions are not evident in the arterial vasculature until a level of chronic inflammation is reached^38^,^39^. Panx1 channels, therefore, may provide a regulated mechanism by which physiological homing of inflammatory cells through the venous endothelium is tightly regulated.

Here, we have characterized a molecular signalling pathway involving activation of type-1 TNF receptors, SFK and phosphorylation of Panx1 channels. Recent evidence has identified a novel role for TNFR1 as a pseudo-receptor tyrosine kinase, achieved through docking of Src kinase to an intracellular domains of the receptor^29^,^30^. Moreover, the regulation of Panx1 activity by kinase-driven signalling mechanisms is gaining support. Specifically, a prominent role for SFK in promoting Panx1 channel activity has been established in neurons during anoxic depolarizations in ischaemia^32^. However, to date there has been no direct evidence for Panx1 phosphorylation. Our analysis of SFK-dependent Panx1 activation revealed that these channels are indeed phosphorylated and that this modification may promote an open-channel state. While the precise mode of Panx1 activation in response to SFK-mediated phosphorylation is still under investigation, recent reports hypothesize that channel gating is intimately regulated by interactions between the C-terminal tail and the channel pore^40^,^41^. It is interesting to speculate that phosphorylation of the Panx1 intracellular loop at Y198 may impart electrostatic interactions with the C-tail to sequester this region from the pore and promote an open-channel conformation. It also currently remains unclear as to whether Panx1 channels are directly activated by phosphorylation of Y198 or whether additional tyrosine residues are modified contributing to this effect. In addition to Src, the serine/threonine kinases ERK and p38MAPK have been reported to rapidly activate in response to TNF-α stimulation, which may contribute to the regulation of Panx1 channels in the endothelium^42^,^43^. Thus, dynamic phosphorylation of Panx1 may be evident during vascular inflammation and be a factor influencing the heterogeneity in venous versus arterial EC-dependent ATP release.

The kinetics of TNF-α-dependent activation of Panx1 in venous ECs suggests that the channels operate transiently to mediate ATP release and downstream purinergic cascades. Specifically, activation of ECs with TNF-α induced ATP release and YO-PRO-1 dye uptake within minutes with concurrent SFK activation and Panx1 phosphorylation. With respect to the transient nature of Panx1 activity in this response, a number of regulatory mechanisms may be in place to prevent excessive ATP release. ATP itself has been reported to negatively regulate channel activity following accumulation in the extracellular compartment^44^. It is suggested that ATP acts allosterically near the extracellular vestibule of the Panx1 permeation pore to limit further ATP release. Examination of the kinetics of ATP release between our cell culture models and perfused vessels revealed a saturation under static conditions where ATP was allowed to accumulate in the extracellular milieu surrounding cultured cells. However, during continuous lumenal perfusion in our isolated vessel system, ATP continually accumulated in the perfusate, which may be explained by removal of the purine by flow and prevention of an ATP block on Panx1 channels. ECs also harbour an endogenous negative-feedback mechanism to control Panx1 function. Specifically, our lab has reported an inhibitory effect of the EC-derived bioactive gas nitric oxide (NO) on Panx1 activity through targeted S-nitrosylation of two conserved cysteine thiols, one located in the predicted pore-lining region of the channel and one in the C-tail^15^. Targeted S-nitrosylation of these residues reduces ATP release and Panx1 channel currents in murine ECs. Activation of EC purinergic receptors by ATP can increase NO production, which may serve as a possible off switch to prevent cytosolic ATP depletion and loss of ionic gradients controlled by ATP-regulated ion transporters. Src is also regulated by S-nitrosylation with modification reported to increase its kinase activity^45^,^46^. On the basis of these observations, there may be a dynamic interplay between Src and Panx1 S-nitrosylation during inflammation, which could regulate the balance between cell survival pathways and cell death. Of particular interest, the temporal dynamics of Panx1 and Src post-translational modification will aid in determining the potential effects of NO on EC-dependent inflammatory signalling during the acute and chronic phases. In addition, a prominent role for NO as an anti-inflammatory mediator has been well established, where increased NO production in the vasculature reduces leukocyte rolling, adhesion and emigration^47^,^48^,^49^,^50^,^51^,^52^. TNF-α can also induce rapid activation of NADPH oxidase, which may alter the redox state of cysteine residues in Panx1 and modify NO-dependent channel regulation^42^. Future studies will provide useful insight into the regulation of Panx1 channels by oxidative stress and NO during inflammatory signalling; however, it is interesting to speculate that a portion of the anti-inflammatory effects of NO are due to S-nitrosylation of Panx1 to limit the release of pro-inflammatory ATP.

Our *in vitro* and *in vivo* analyses of leukocyte–EC interactions in response to the acute inflammatory stimulus TNF-α identified a major contribution of venous EC Panx1 channels in promoting adhesion to and extravasation through the vascular wall. Blocking Panx1 activity pharmacologically *in vitro*, and molecularly *in vivo*, reduced leukocyte adhesion to TNF-α-primed ECs and abolished emigration of circulating and adherent leukocytes through post-capillary venules in the exteriorized mouse cremaster preparation. This effect was specific to the expression of EC Panx1 channels, since administration of tamoxifen to C57Bl/6 mice had no effect on TNF-α-stimulated adhesion and emigration (Supplementary Fig. 7b,c) as compared with the potent ablation in both processes in VECadER^T2+^/Panx1^fl/fl^ mice, which received tamoxifen. No significant differences were seen in rolling velocity and the absolute number of rolling leukocytes between animals, indicating that the activity of Panx1 channels predominantly contributes to the downstream events including adhesion and emigration. The initial rolling and attachment of inflammatory cells to the endothelium is controlled primarily by selectin molecules (P-selectin and E-selectin), while firm adhesion and extravasation is promoted by the upregulation of VCAM1 and ICAM1. In accordance with this, we observed that deletion of Panx1 from the endothelium prevented TNF-α-mediated VCAM1 upregulation implicating Panx1-dependent ATP release in the latter phase of inflammatory cell homing. This effect could be rescued by the exogenous application of ATP. These experiments link previous reports of purinergic signalling through P2Y receptors in the acute inflammatory cascade with Panx1-mediated release of ATP from the endothelium. While our investigation focused on the role of EC Panx1 channels and the role of released ATP on EC phenotype, leukocytes also use purinergic signalling for activation during inflammation^53^,^54^,^55^. It is now evident that neutrophils release ATP in response to activation by danger signals. In particular, activation of neutrophils with fMLP promotes ATP release in part via Panx1 channels, which signals in an autocrine fashion to activate P2Y2 receptors and promote chemotaxis^56^. Neutrophils also express TNF receptors; however, whether activation of these receptors induces Panx1 activation or ATP released from EC Panx1 channels contributes to neutrophil chemotaxis has not been investigated. Nonetheless, it is possible that the reduced interactions observed between endogenous leukocytes and the venous endothelium in EC Panx1 KO mice may reflect diminished purinergic signalling in both ECs and the inflammatory cells. Taken together, the results presented in this study highlight a novel role for EC Panx1 channels in the regulation of acute vascular inflammation, poising Panx1 as a linking factor between TNF-α signalling and purinergic control of inflammatory cell interactions with the blood vessel wall.

## Methods

### Cell culture

Primary HUVECs were purchased from Cell Applications (200K-05). Primary HSaVECs and HAoECs were from PromoCell (C-12231 and C-12271, respectively) and HCoAECs were purchased from Lonza (CC-2585). All ECs were maintained under standard cell culture conditions in endothelial growth medium (EGM-2MV) from Lonza. For siRNA knockdown of Panx1 and Cx43, HUVEC and HSaVECs were plated in six-well (expression) or 24-well plates (ATP release) and grown to 70–80% confluence. Non-targeting siRNAs (Life Technologies silencer select negative control 1, 4390843) or siRNAs targeting the human *PANX1* gene (Life Technologies Panx1 silencer select 4392420-s24427; 5′-GUUUGUGGGAGGUAUCUGAtt-3′) or *Cx43* (Life Technologies GJA1 silencer select 4392420-s5758; 5′-GGCUAAUUACAGUGCAGAAtt-3′) were transfected into ECs using Lipofectamine RNAiMAX reagent, and knockdown was assessed via western blotting following a 72-h incubation.

### Mice

All mice were male, 10–14 weeks of age, on a C57Bl/6J genetic background, and were cared for under the provisions of the University of Virginia Animal Care and Use Committee and the LSU Health Sciences Center-Shreveport Animal Care and Use Committee and followed the National Institute of Health guidelines for the care and use of laboratory animals. The inducible, EC-specific Panx1 KO mice (*VECadER*^*T2+*^*/Panx1*^*fl/fl*^) were generated by crossing *VECadER*^*T2+*^*/Panx1*^*WT/WT*^ mice (a kind gift from Dr Ralf Adams, Max Plank Institute, Germany) with *VECadER*^*T2−*^*/Panx1*^*fl/fl*^ mice^28^. To selectively induce Panx1 deletion in the vascular endothelium, *VECadER*^*T2+*^*/Panx1*^*fl/fl*^ mice received intraperitoneal injections of Tamoxifen (1 mg in 0.1 ml PO) for 10 consecutive days. A subset of *VECadER*^*T2+*^*/Panx1*^*fl/fl*^ mice were injected with PO (the vehicle for tamoxifen) and served as littermate controls.

### ATP release assays

*In vitro*. Human arterial and venous ECs were seeded in 24-well plates pre-coated with 0.2% gelatin and grown to confluency. On the day of the experiment, the media was removed from each well and cells were carefully washed twice with warm serum-free basal EC medium supplemented with 1% BSA. Cells were then incubated in 300 μl of fresh basal EC medium supplemented with 1% BSA for 30 min at 37 °C to allow degradation of extracellular ATP released due to mechanical stimulation during washes. Endogenous ecto-nucleotidases were inhibited by incubating EC monolayers with 300 μM ARL 67156 (Tocris) for 30 min at 37 °C. Cells were then stimulated with recombinant human TNF-α (R&D Systems). For dose–response experiments, ECs were incubated with 0.1, 1, 5, 10, 50 or 100 ng ml^−1^ recombinant TNF-α for 30 min at 37 °C. For time-course experiments, ECs were stimulated with 10 ng ml^−1^ recombinant TNF-α for different time points up to 1 h. In experiments where pharmacological inhibitors were employed, ECs were incubated with antagonists in parallel with ARL 67156 for 30 min. To inhibit TNFR1, ECs were incubated with the peptide antagonist of the receptor WP9QY (10 μM; Anaspec). Vesicular ATP release was inhibited with BFA (5 μg ml^−1^; Sigma) and CALHM1 channels were pharmacologically blocked with ruthenium red (20 μM; Sigma). Cx hemichannels were inhibited with lanthanum (La^3+^; 100 μM; Sigma) and Panx1 channels were blocked with CBX (50 μM; Sigma) and the inhibitory peptide ^10^panx1 (200 μM; Genscript). To assess the role of SFK, cells were incubated with the SFK inhibitor PP2 (10 μM; Tocris) or its inactive analogue PP3 (10 μM; Tocris). The role of caspase activation was assessed by inhibition with the pan-caspase blocker Q-VD-OPh (100 μM; Sigma). Following stimulation with TNF-α or vehicle, 150 μl of the cell supernatant was collected and immediately placed into pre-chilled 1.5 ml Eppendorf tubes on ice. All samples were centrifuged at 10,000*g* for 5 min and 50 μl of each sample was transferred to a white, opaque 96-well plate. Using a FluoStar Omega luminometer, 50 μl of luciferin:luciferase reagent (ATP bioluminescence assay kit HSII; Roche) was injected into each well and luminescence was recorded following a 5-s orbital mix. ATP concentration in each sample was calculated from a standard curve for all experiments. Data are presented as a % change in ATP release from baseline (that is, unstimulated cells) and expressed as mean±s.e.m. (*n*=5 independent experiments with triplicate measurements).

### *Ex vivo*

For isolated blood vessel experiments, seconed-order venules or arterioles were dissected from the mouse mesentery circulation and cannulated on glass micropipettes to access the vessel lumen. Following cannulation, vessels were perfused lumenally with a MOPS-buffered physiological salt solution (NaCl 145 mM, KCl 4.7 mM, CaCl_2_ 2 mM, MgSO_4_ 1.17 mM, NaH_2_PO_4_ 1.2 mM, glucose 5 mM, pyruvate 2 mM, EDTA 0.02 mM and MOPS 2 mM) to remove red blood cells and equilibrated at 37 °C for 20 min. Vessels were then perfused with MOPS PSS containing 300 μM ARL 67156 +/− pharmacological inhibitors and incubated for an additional 20 min prior to perfusion with MOPS PSS containing recombinant murine TNF-α (R&D Systems). Aliquots of the lumenal perfusate were collected every 5 min for a 25-min period, and the ATP concentration was quantified by bioluminescence as described above. Data are expressed as mean±s.e.m. (*n*=4 independent experiments).

### LDH release assays

Isolated mesenteric venules were cannulated and perfused with recombinant mouse TNF-α for 20 min followed by lysis buffer. Perfusate samples were collected as described above and LDH activity was assayed with the Cytotoxicity Detection Kit Plus (Roche) according to the manufacturer’s protocol as an output for cell death. Data are expressed as mean±s.e.m. (*n*=3 independent experiments).

### YO-PRO-1 dye uptake assays

HUVECs were cultured on poly-L-lysine-coated coverslips and grown to confluence in EGM. Cells were washed three times with 1 × PBS and incubated in fresh EGM for 30 min prior to stimulation. To stimulate YO-PRO-1 dye uptake, YO-PRO-1 (1 μM) was added to the media and EC monolayers were treated with TNF-α at a range of concentrations (0.1–100 ng ml^−1^) for 30 min. At the end of the stimulation, cells were washed 3 × with 1 × PBS and immediately fixed in 4% paraformaldehyde (PFA). Coverslips were then mounted on microscope slides with DAPI Prolong Gold mounting agent and visualized on an Olympus IX81 laser scanning confocal microscope. Dye uptake was quantified as the intensity of YO-PRO-1 fluorescence over time using ImageJ software. For time-course analysis, YO-PRO-1 was added to the culture media at various time points after TNF-α stimulation and incubated to the 30-min time point. Data are expressed as mean±s.e.m. (*n*=3 independent experiments).

### Connexin hemichannel depletion/cell surface biotinylation

Cell surface biotinylation was performed as described previously^15^. To deplete Cx43 hemichannels from the EC plasma membrane, venous and arterial ECs were grown to confluency in six-well plates (western blotting) or 24-well plates (ATP release) and treated with the vesicular exocytosis inhibitor BFA (5 μg ml^−1^) for 5 h at 37 °C. Cells were washed once with cold 1 × PBS and incubated with cold DMEM (without FBS) and 50 μM CBX at 4 °C for 30 min. CBX was added to prevent biotin from passing through Panx1 channels, which may label intracellular proteins. Cells were washed with PBS and incubated at 4 °C for 1 h in cold PBS (1.5 ml per dish) containing EZ-link-sulfo-NHS-LC-biotin (1 mg ml^−1^) and CBX (50 μM). The cells were washed again with PBS and lysed in PBS-T (PBS+0.5–1% Triton-X 100) containing protease inhibitors. Total protein was quantified using the bicinchoninic (BCA) assay and equal amounts of protein were incubated with streptavidin-agarose beads for 2 h at 4 °C to pull down biotinylated (cell surface) proteins. Beads were then washed five times with 1 × PBS-T, pelleted by centrifugation and proteins eluted by incubation with 5 × sample buffer. Eluted proteins were subjected to SDS–polyacrylamide gel electrophoresis and western blotting for detection of Cx43 and Panx1.

### Immunofluorescence microscopy

Male *VECadER*^*T2+*^*/Panx1*^*fl/fl*^ mice (tamoxifen or PO injected) were euthanized by CO_2_ asphyxiation and subsequent cervical dislocation. Prior to tissue harvesting, fixation was performed by perfusing room temperature 4% PFA made in PBS through the heart. The mesentery was immediately excised and second-order venules were dissected free of surrounding fat and connective tissue and placed in 4% PFA for 30 min before transfer to 70% ethanol for paraffin embedding. Paraffin sections (4–5 μm in thickness) were deparaffinized and processed for immunocytochemistry as previously described^10^. For validation of Panx1 KO in the endothelium, vessel sections were processed for conventional immunolabelling and incubated overnight at 4 °C with a primary antibody directed against the murine Panx1 C-tail^26^. To analyse VCAM1 expression, a polyclonal antibody to murine VCAM1 was used (Abcam; 1:500).

### Immuno-transmission electron microscopy (iTEM)

This was performed as previously described, using an extracellular loop Panx1 antibody^11^. Gold beads were pseudo-coloured pink for visualization.

### Western blotting for Src-family kinase/Panx1 phosphorylation

Following stimulation with TNF-α (*in vitro*: 10 ng ml^−1^; *ex vivo*: 50 ng ml^−1^), confluent EC monolayers or isolated mesenteric venules were homogenized in ice-cold Triton extraction buffer (50 mM Tris-HCL, 150 mM NaCl, 5 mM EDTA, 1% deoxycholate, 1% NP-40 and 1% Triton-X100 in PBS and pH adjusted to 7.4) containing protease and phosphatase inhibitors. Cell/tissue lysates were incubated with rotation at 4 °C for 20 min to solubilize proteins, followed by centrifugation for 5 min at 13,000*g* to pellet cell debris. Protein concentration was quantified using the BCA method. In all, 15–20 μg of total protein was subjected to SDS gel electrophoresis using 4–12% Bis-Tris gels (Invitrogen) and transferred to nitrocellulose membranes for immunoblotting. Membranes were blocked for 1 h at room temperature with LiCOR blocking solution, then incubated overnight at 4 °C with primary antibodies against pY416SFKs (BD Biosciences; 1:1,000), pY198Panx1 (Alpha Diagnostic Intl (ADI); 1:1,000) and GAPDH (Sigma; 1:10,000) or an antibody against the intracellular loop of Panx1 (Panx1-IL) (ADI; 1:1,000) (normalization controls). Membranes were then washed and incubated with LiCOR secondary antibodies (1:10,000) and visualized and quantified using LiCOR Odyssey. To inhibit SFK activity, EC monolayers were incubated with the SFK inhibitor PP2 (Tocris; 10 μM). The inactive analogue PP3 (Tocris; 10 μM) was used as a negative control for PP2. To show specificity for the phosphorylated form of SFKs and Panx1, cell lysates were incubated with alkaline phosphatase for 1 h at 37 °C. Western blot images have been cropped for presentation. Uncropped western blots can be found in Supplementary Figs 8 and 9. Data are expressed as mean±s.e.m. (*n*=3 independent experiments).

### Static adhesion assay

HUVECs were seeded on gelatin-coated glass coverslips in six-well plates and grown to confluence. On the day of the experiment, cells were washed 2 × with 1 × PBS and incubated for 30 min in basal EGM supplemented with 1% BSA in the presence or absence of pharmacological inhibitors. During this incubation period, human THP-1 monocytes were loaded with Calcein-AM (Invitrogen; 5 μg ml^−1^) for 30 min at 37 °C. Cells were then pelleted by brief centrifugation and excess calcein was removed by washing THP-1 cells three times in 1 × PBS. Next, HUVECs were stimulated with TNF-α (10 ng ml^−1^) for 30 min. Following EC activation, calcein-loaded THP-1 monocytes were added to EC monolayers and incubated for 20 min at 37 °C to allow THP-1 to contact ECs. Monolayers were then washed 3 × with 1 × PBS and fixed in ice-cold 4% PFA. Following fixation, coverslips were removed from the six-well plates and mounted on glass microscope slides with Prolong Gold anti-fade mountant with DAPI (Life Technologies). Fluorescent micrographs were obtained at × 10 magnification and the number of adherent THP-1 monocytes/ECs was quantified in five randomized regions per slide. Data are expressed as mean±s.e.m. (*n*=3 independent experiments).

### Intravital microscopy for leukocyte adhesion and emigration

Mice were prepared for intravital microscopy of the cremaster muscle as described previously^57^. Briefly, mice were anaesthetized with ketamine hydrochloride (150 mg kg^−1^) and xylazine (7.5 mg kg^−1^), intraperitoneally. The cremaster was isolated, laid over a viewing pedestal, superfused with bicarbonate-buffered saline. After 30 min equilibration, a venule with a wall shear rate of ≥500 s^−1^, diameter between 20 and 40 μm and the least number of adherent and emigrated leukocytes was chosen for further study. A 1-min baseline recording was made, after which superfusion was stopped, and 50 μl TNF-α (1.7 ng ml^−1^ in 0.1% BSA) or vehicle control was added under a saran wrap cover every 30 min. One-minute recordings were made just prior to each addition, for 3 h. The TNF-α was initially reconstituted in PBS at 3.4 ng ml^−1^ and 150-μl aliquots were frozen. An aliquot was mixed 1:1 with BBS containing 0.2% BSA at the time of the experiment. Leukocyte adhesion and emigration were determined by off-line analysis. Leukocytes were considered adherent if they stopped for at least 30 s (expressed as number of cells per mm^2^ vessel wall), and emigrated leukocytes were leukocytes identified in the interstitium (expressed as number of cells per mm^2^ tissue). Data are expressed as mean±s.e.m. (*n*=6–7 independent animals per group).

### Statistics

All statistics were performed using GraphPad Prizm software. For multiple comparisons, statistics were performed using a one-way or two-way analysis of variance followed by pairwise analysis. A Student’s *t*-test was used for individual comparisons if normally distributed.

## Additional information

**How to cite this article:** Lohman, A. W. *et al.* Pannexin 1 channels regulate leukocyte emigration through the venous endothelium during acute inflammation. *Nat. Commun.* 6:7965 doi: 10.1038/ncomms8965 (2015).

## Supplementary Material

Supplementary Information Supplementary Figures 1-9

## References

[b1] Ley K., Laudanna C., Cybulsky M. I. & Nourshargh S. Getting to the site of inflammation: the leukocyte adhesion cascade updated. Nat. Rev. Immunol. 7, 678–689 (2007). 17717539 10.1038/nri2156

[b2] Ralevic V. & Burnstock G. Receptors for purines and pyrimidines. Pharmacol. Rev. 50, 413–492 (1998). 9755289

[b3] Zerr M. *et al.* Major contribution of the P2Y(1)receptor in purinergic regulation of TNFalpha-induced vascular inflammation. Circulation 123, 2404–2413 (2011). 21576651 10.1161/CIRCULATIONAHA.110.002139

[b4] Riegel A. K. *et al.* Selective induction of endothelial P2Y6 nucleotide receptor promotes vascular inflammation. Blood 117, 2548–2555 (2011). 21173118 10.1182/blood-2010-10-313957 PMC3062416

[b5] Hyman M. C. *et al.* Self-regulation of inflammatory cell trafficking in mice by the leukocyte surface apyrase CD39. J. Clin. Invest. 119, 1136–1149 (2009). 19381014 10.1172/JCI36433 PMC2673847

[b6] McDonald B. *et al.* Intravascular danger signals guide neutrophils to sites of sterile inflammation. Science 330, 362–366 (2010). 20947763 10.1126/science.1195491

[b7] Reutershan J. *et al.* Adenosine and inflammation: CD39 and CD73 are critical mediators in LPS-induced PMN trafficking into the lungs. FASEB J. 23, 473–482 (2009). 18838482 10.1096/fj.08-119701

[b8] Bouma M. G., van den Wildenberg F. A. & Buurman W. A. Adenosine inhibits cytokine release and expression of adhesion molecules by activated human endothelial cells. Am. J. Physiol. 270, C522–C529 (1996). 8779915 10.1152/ajpcell.1996.270.2.C522

[b9] Koszalka P. *et al.* Targeted disruption of cd73/ecto-5'-nucleotidase alters thromboregulation and augments vascular inflammatory response. Circ. Res. 95, 814–821 (2004). 15358667 10.1161/01.RES.0000144796.82787.6f

[b10] Lohman A. W. *et al.* Expression of pannexin isoforms in the systemic murine arterial network. J. Vasc. Res. 49, 405–416 (2012). 22739252 10.1159/000338758 PMC3482510

[b11] Billaud M. *et al.* Pannexin1 regulates alpha1-adrenergic receptor- mediated vasoconstriction. Circ. Res. 109, 80–85 (2011). 21546608 10.1161/CIRCRESAHA.110.237594 PMC3135971

[b12] Gaynullina D., Shestopalov V. I., Panchin Y. & Tarasova O. S. Pannexin 1 facilitates arterial relaxation via an endothelium-derived hyperpolarization mechanism. FEBS Lett. 589, 1164–1170 (2015). 25819435 10.1016/j.febslet.2015.03.018

[b13] Gaynullina D., Tarasova O. S., Kiryukhina O. O., Shestopalov V. I. & Panchin Y. Endothelial function is impaired in conduit arteries of pannexin1 knockout mice. Biol. Direct 9, 8 (2014). 24885326 10.1186/1745-6150-9-8 PMC4046076

[b14] Bao L., Locovei S. & Dahl G. Pannexin membrane channels are mechanosensitive conduits for ATP. FEBS Lett. 572, 65–68 (2004). 15304325 10.1016/j.febslet.2004.07.009

[b15] Lohman A. W. *et al.* S-nitrosylation inhibits pannexin 1 channel function. J. Biol. Chem. 287, 39602–39612 (2012). 23033481 10.1074/jbc.M112.397976 PMC3501028

[b16] Adamson S. E. & Leitinger N. The role of pannexin1 in the induction and resolution of inflammation. FEBS Lett. 588, 1416–1422 (2014). 24642372 10.1016/j.febslet.2014.03.009 PMC4060616

[b17] Pelegrin P. & Surprenant A. Pannexin-1 mediates large pore formation and interleukin-1beta release by the ATP-gated P2X7 receptor. EMBO J. 25, 5071–5082 (2006). 17036048 10.1038/sj.emboj.7601378 PMC1630421

[b18] Silverman W. R. *et al.* The pannexin 1 channel activates the inflammasome in neurons and astrocytes. J. Biol. Chem. 284, 18143–18151 (2009). 19416975 10.1074/jbc.M109.004804 PMC2709345

[b19] Chekeni F. B. *et al.* Pannexin 1 channels mediate 'find-me' signal release and membrane permeability during apoptosis. Nature 467, 863–867 (2010). 20944749 10.1038/nature09413 PMC3006164

[b20] Woehrle T. *et al.* Pannexin-1 hemichannel-mediated ATP release together with P2X1 and P2X4 receptors regulate T-cell activation at the immune synapse. Blood 116, 3475–3484 (2010). 20660288 10.1182/blood-2010-04-277707 PMC2981474

[b21] Gulbransen B. D. *et al.* Activation of neuronal P2X7 receptor-pannexin-1 mediates death of enteric neurons during colitis. Nat. Med. 18, 600–604 (2012). 22426419 10.1038/nm.2679 PMC3321107

[b22] Riteau N. *et al.* Extracellular ATP is a danger signal activating P2X7 receptor in lung inflammation and fibrosis. Am. J. Respir. Crit. Care Med. 182, 774–783 (2010). 20522787 10.1164/rccm.201003-0359OC

[b23] Lohman A. W., Billaud M. & Isakson B. E. Mechanisms of ATP release and signalling in the blood vessel wall. Cardiovasc. Res. 95, 269–280 (2012). 22678409 10.1093/cvr/cvs187 PMC3400358

[b24] Lohman A. W. & Isakson B. E. Differentiating connexin hemichannels and pannexin channels in cellular ATP release. FEBS Lett. 588, 1379–1388 (2014). 24548565 10.1016/j.febslet.2014.02.004 PMC3996918

[b25] Laird D. W. Life cycle of connexins in health and disease. Biochem. J. 394, 527–543 (2006). 16492141 10.1042/BJ20051922 PMC1383703

[b26] Penuela S. *et al.* Pannexin 1 and pannexin 3 are glycoproteins that exhibit many distinct characteristics from the connexin family of gap junction proteins. J. Cell Sci. 120, 3772–3783 (2007). 17925379 10.1242/jcs.009514

[b27] Taruno A. *et al.* CALHM1 ion channel mediates purinergic neurotransmission of sweet, bitter and umami tastes. Nature 495, 223–226 (2013). 23467090 10.1038/nature11906 PMC3600154

[b28] Poon I. K. *et al.* Unexpected link between an antibiotic, pannexin channels and apoptosis. Nature 507, 329–334 (2014). 24646995 10.1038/nature13147 PMC4078991

[b29] Pincheira R., Castro A. F., Ozes O. N., Idumalla P. S. & Donner D. B. Type 1 TNF receptor forms a complex with and uses Jak2 and c-Src to selectively engage signaling pathways that regulate transcription factor activity. J. Immunol. 181, 1288–1298 (2008). 18606683 10.4049/jimmunol.181.2.1288

[b30] Xing L. *et al.* Genetic evidence for a role for Src family kinases in TNF family receptor signaling and cell survival. Genes Dev. 15, 241–253 (2001). 11157779 10.1101/gad.840301 PMC312612

[b31] Okutani D., Lodyga M., Han B. & Liu M. Src protein tyrosine kinase family and acute inflammatory responses. Am. J. Physiol. Lung Cell. Mol. Physiol. 291, L129–L141 (2006). 16581827 10.1152/ajplung.00261.2005

[b32] Weilinger N. L., Tang P. L. & Thompson R. J. Anoxia-induced NMDA receptor activation opens pannexin channels via Src family kinases. J. Neurosci. 32, 12579–12588 (2012). 22956847 10.1523/JNEUROSCI.1267-12.2012 PMC6621249

[b33] Kmiecik T. E. & Shalloway D. Activation and suppression of pp60c-src transforming ability by mutation of its primary sites of tyrosine phosphorylation. Cell 49, 65–73 (1987). 3103925 10.1016/0092-8674(87)90756-2

[b34] Billaud M. *et al.* A molecular signature in the pannexin1 intracellular loop confers channel activation by the alpha1 adrenoreceptor in smooth muscle cells. Sci. Signal. 8, ra17 (2015). 25690012 10.1126/scisignal.2005824 PMC4358815

[b35] Baker O. J., Camden J. M., Rome D. E., Seye C. I. & Weisman G. A. P2Y2 nucleotide receptor activation up-regulates vascular cell adhesion molecule-1 [corrected] expression and enhances lymphocyte adherence to a human submandibular gland cell line. Mol. Immunol. 45, 65–75 (2008). 17599409 10.1016/j.molimm.2007.05.009 PMC2064040

[b36] Smedlund K. & Vazquez G. Involvement of native TRPC3 proteins in ATP-dependent expression of VCAM-1 and monocyte adherence in coronary artery endothelial cells. Arterioscler. Thromb. Vasc. Biol. 28, 2049–2055 (2008). 18787184 10.1161/ATVBAHA.108.175356

[b37] Vanderstocken G. *et al.* P2Y2 receptor regulates VCAM-1 membrane and soluble forms and eosinophil accumulation during lung inflammation. J. Immunol. 185, 3702–3707 (2010). 20720203 10.4049/jimmunol.0903908

[b38] Marchesi V. T. The site of leucocyte emigration during inflammation. Q. J. Exp. Physiol. Cogn. Med. Sci. 46, 115–118 (1961). 13766496 10.1113/expphysiol.1961.sp001522

[b39] Marchesi V. T. & Florey H. W. Electron micrographic observations on the emigration of leucocytes. Q. J. Exp. Physiol. Cogn. Med. Sci. 45, 343–348 (1960). 13766495 10.1113/expphysiol.1960.sp001489

[b40] Sandilos J. K. *et al.* Pannexin 1, an ATP release channel, is activated by caspase cleavage of its pore-associated C-terminal autoinhibitory region. J. Biol. Chem. 287, 11303–11311 (2012). 22311983 10.1074/jbc.M111.323378 PMC3322839

[b41] Dourado M., Wong E. & Hackos D. H. Pannexin-1 is blocked by its C-terminus through a delocalized non-specific interaction surface. PLoS ONE 9, e99596 (2014). 24911976 10.1371/journal.pone.0099596 PMC4049774

[b42] Li J. M., Fan L. M., Christie M. R. & Shah A. M. Acute tumor necrosis factor alpha signaling via NADPH oxidase in microvascular endothelial cells: role of p47phox phosphorylation and binding to TRAF4. Mol. Cell Biol. 25, 2320–2330 (2005). 15743827 10.1128/MCB.25.6.2320-2330.2005 PMC1061612

[b43] Marques-Fernandez F. *et al.* TNFalpha induces survival through the FLIP-L-dependent activation of the MAPK/ERK pathway. Cell Death Dis. 4, e493 (2013). 23412386 10.1038/cddis.2013.25 PMC3734812

[b44] Qiu F. & Dahl G. A permeant regulating its permeation pore: inhibition of pannexin 1 channels by ATP. Am. J. Physiol. Cell Physiol. 296, C250–C255 (2009). 18945939 10.1152/ajpcell.00433.2008 PMC2643853

[b45] Akhand A. A. *et al.* Nitric oxide controls src kinase activity through a sulfhydryl group modification-mediated Tyr-527-independent and Tyr-416-linked mechanism. J. Biol. Chem. 274, 25821–25826 (1999). 10464322 10.1074/jbc.274.36.25821

[b46] Rahman M. A. *et al.* S-nitrosylation at cysteine 498 of c-Src tyrosine kinase regulates nitric oxide-mediated cell invasion. J. Biol. Chem. 285, 3806–3814 (2010). 19948721 10.1074/jbc.M109.059782 PMC2823522

[b47] VanUffelen B. E., de Koster B. M., Van den Broek P. J., VanSteveninck J. & Elferink J. G. Modulation of neutrophil migration by exogenous gaseous nitric oxide. J. Leukoc. Biol. 60, 94–100 (1996). 8699130 10.1002/jlb.60.1.94

[b48] Kubes P., Suzuki M. & Granger D. N. Nitric oxide: an endogenous modulator of leukocyte adhesion. Proc. Natl Acad. Sci. USA 88, 4651–4655 (1991). 1675786 10.1073/pnas.88.11.4651 PMC51723

[b49] Kubes P. & Granger D. N. Nitric oxide modulates microvascular permeability. Am. J. Physiol. 262, H611–H615 (1992). 1539722 10.1152/ajpheart.1992.262.2.H611

[b50] Dal Secco D. *et al.* Neutrophil migration in inflammation: nitric oxide inhibits rolling, adhesion and induces apoptosis. Nitric Oxide 9, 153–164 (2003). 14732339 10.1016/j.niox.2003.11.001

[b51] Clark S. C., Shenton B. K., Dark J. H. & Kirby J. A. Neutrophil transmigration: modulation by pentoxifylline and nitric oxide. Biochem. Soc. Trans. 25, 454S (1997). 9388678 10.1042/bst025454s

[b52] Chello M., Mastroroberto P., Perticone F., Celi V. & Colonna A. Nitric oxide modulation of neutrophil-endothelium interaction: difference between arterial and venous coronary bypass grafts. J. Am. Coll. Cardiol. 31, 823–826 (1998). 9525554 10.1016/s0735-1097(97)00560-3

[b53] Grassi F. Purinergic control of neutrophil activation. J. Mol. Cell Biol. 2, 176–177 (2010). 20671113 10.1093/jmcb/mjq014

[b54] Ayata C. K. *et al.* Purinergic P2Y(2) receptors promote neutrophil infiltration and hepatocyte death in mice with acute liver injury. Gastroenterology 143, 1620–1629 e1624 (2012). 22974709 10.1053/j.gastro.2012.08.049

[b55] Chen Y. *et al.* ATP release guides neutrophil chemotaxis via P2Y2 and A3 receptors. Science 314, 1792–1795 (2006). 17170310 10.1126/science.1132559

[b56] Bao Y., Chen Y., Ledderose C., Li L. & Junger W. G. Pannexin 1 channels link chemoattractant receptor signaling to local excitation and global inhibition responses at the front and back of polarized neutrophils. J. Biol. Chem. 288, 22650–22657 (2013). 23798685 10.1074/jbc.M113.476283 PMC3829350

[b57] Stokes K. Y., Calahan L., Russell J. M., Gurwara S. & Granger D. N. Role of platelets in hypercholesterolemia-induced leukocyte recruitment and arteriolar dysfunction. Microcirculation 13, 377–388 (2006). 16815823 10.1080/10739680600745877

