# Supplementary material for: Pannexin 1 channels regulate leukocyte emigration through the venous endothelium during acute inflammation
Source: Nat Commun. 2015 Aug 5;6:7965. doi: 10.1038/ncomms8965 (PMC4824045; doi:10.1038/ncomms8965)
Supplement: Supplementary Information — Supplementary Figures 1-9 [file ncomms8965-s1.pdf]

Supplementary Figures and Legends:

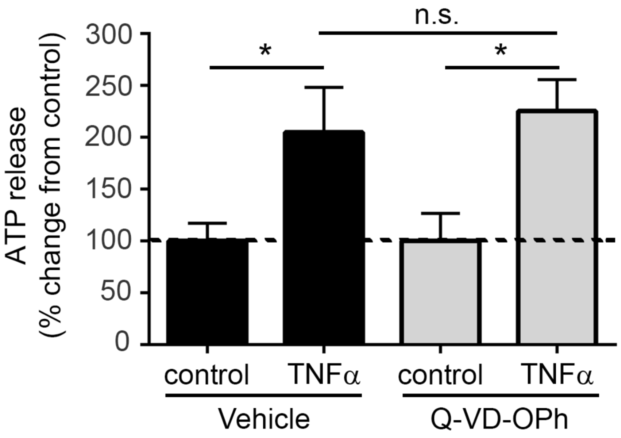

Supplementary Figure 1: TNFα induces ATP release from HUVEC and HSaVEC independent caspase activation (a) TNFα induced ATP release from HUVEC in the presence of the pan-caspase inhibitor QVD-OPh (100 μM). Cells were treated with recombinant human TNFα (100ng/mL) for 30 minutes. *=p<0.05 as compared to unstimulated controls by One-way ANOVA (n=4). Data are presented as mean±SEM (error bars).

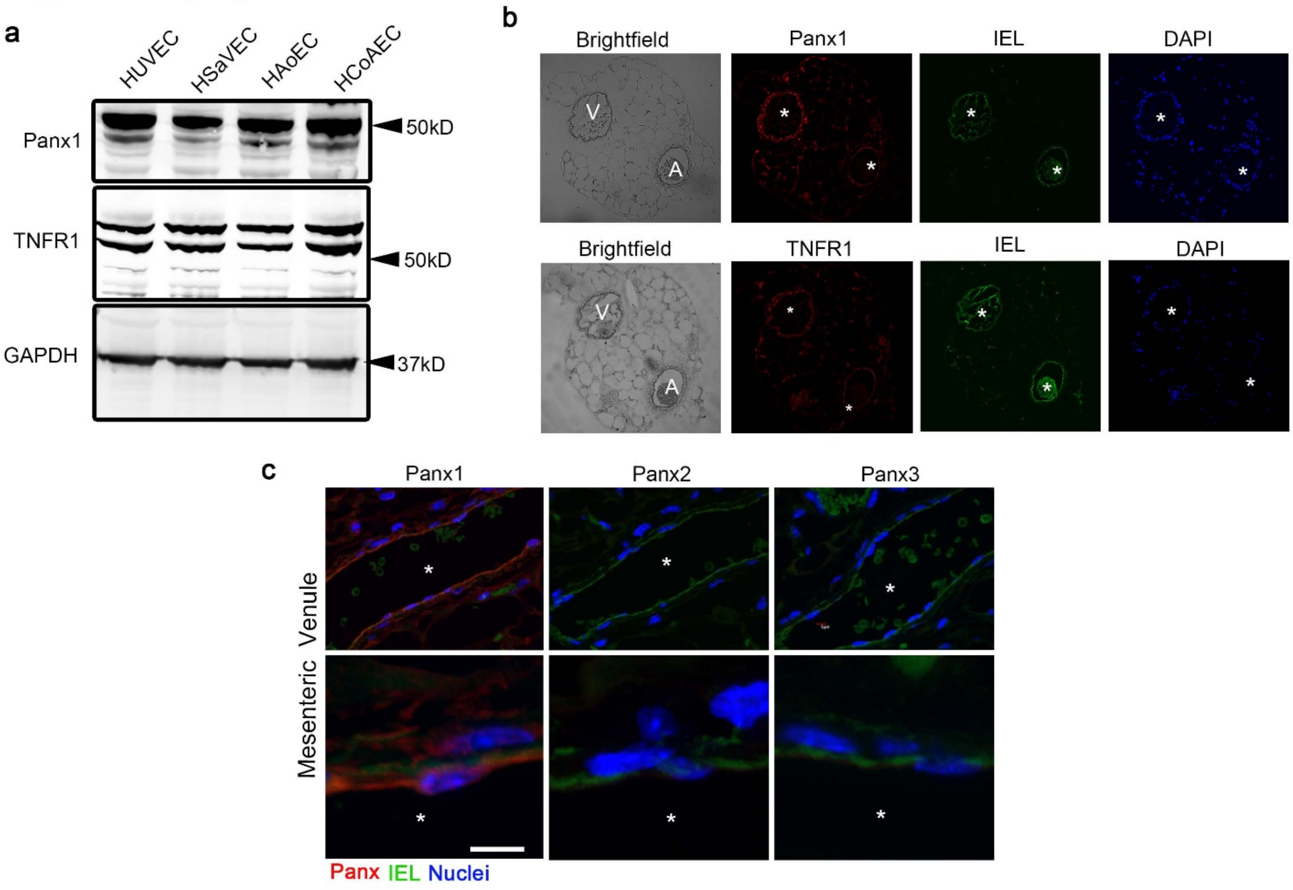

Supplementary Figure 2: TNFR1 and Panx1 expression do not differ between venous and arterial ECs (a) Western blot of Panx1 and TNFR1 expression in primary human venous (HUVEC, HSaVEC) and arterial (HAoEC, HCoAEC) endothelial cells. **(b)** Immunofluorescence micrographs of Panx1 and TNFR1 expression in isolated mesenteric arteriole (A) and venule (V) pairs. * indicate the vessel lumen, autofluorescence of the IEL is in green and nuclei are stained with DAPI (blue). **(c)** Panx 1, 2 and 3 expression profile in isolated mesenteric venules. Panx expression is in red, autofluorescence of the IEL is in green and nuclei are stained with DAPI (blue).

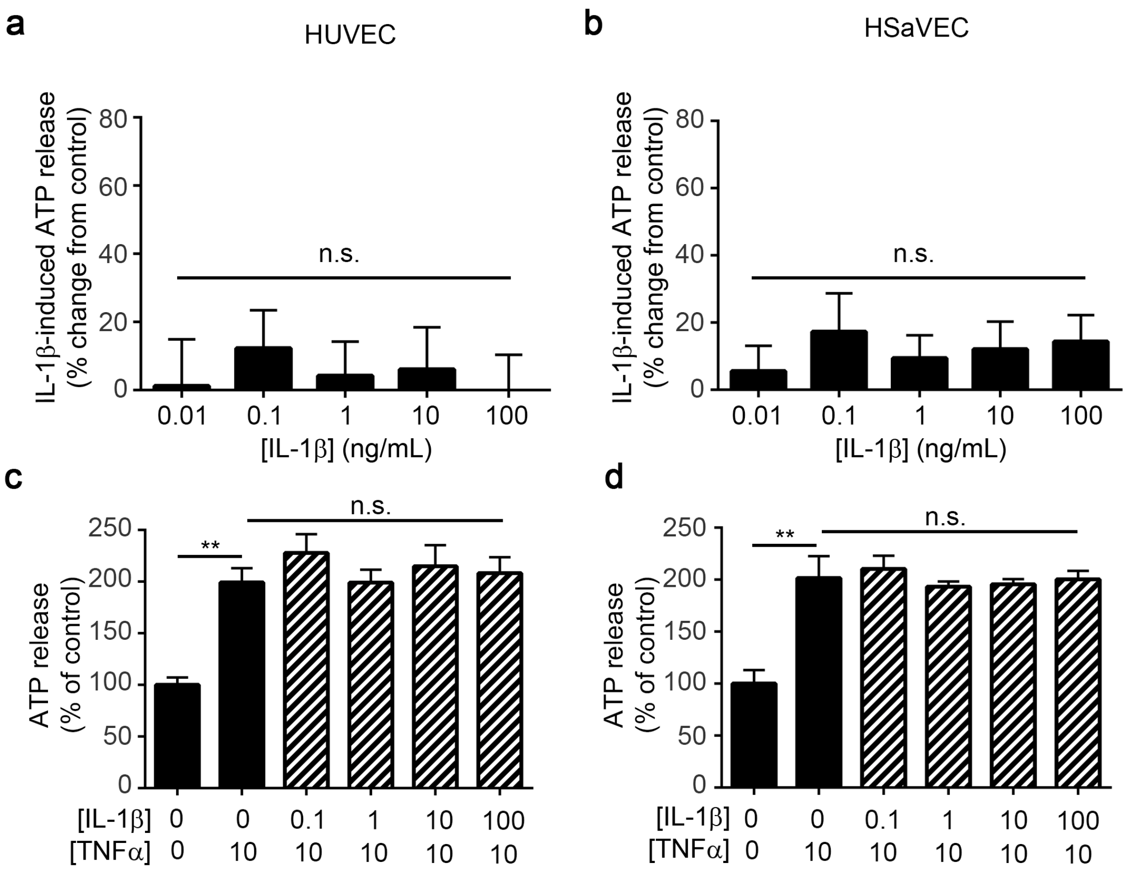

Supplementary Figure 3: IL-1β does not promote ATP release from HUVEC or HSaVECs (a-b) ATP release from HUVEC **(a)** and HSaVEC **(b)** to increasing concentrations of recombinant human IL-1β. **(c-d)** ATP release from HUVEC **(c)** and HSaVEC **(d)** to co-stimulation with TNFα (10ng/mL) and increasing concentrations of IL-1β. **=$p<0.01$ compared to unstimulated controls by One-way ANOVA (n=4). All data are presented as mean±SEM (error bars).

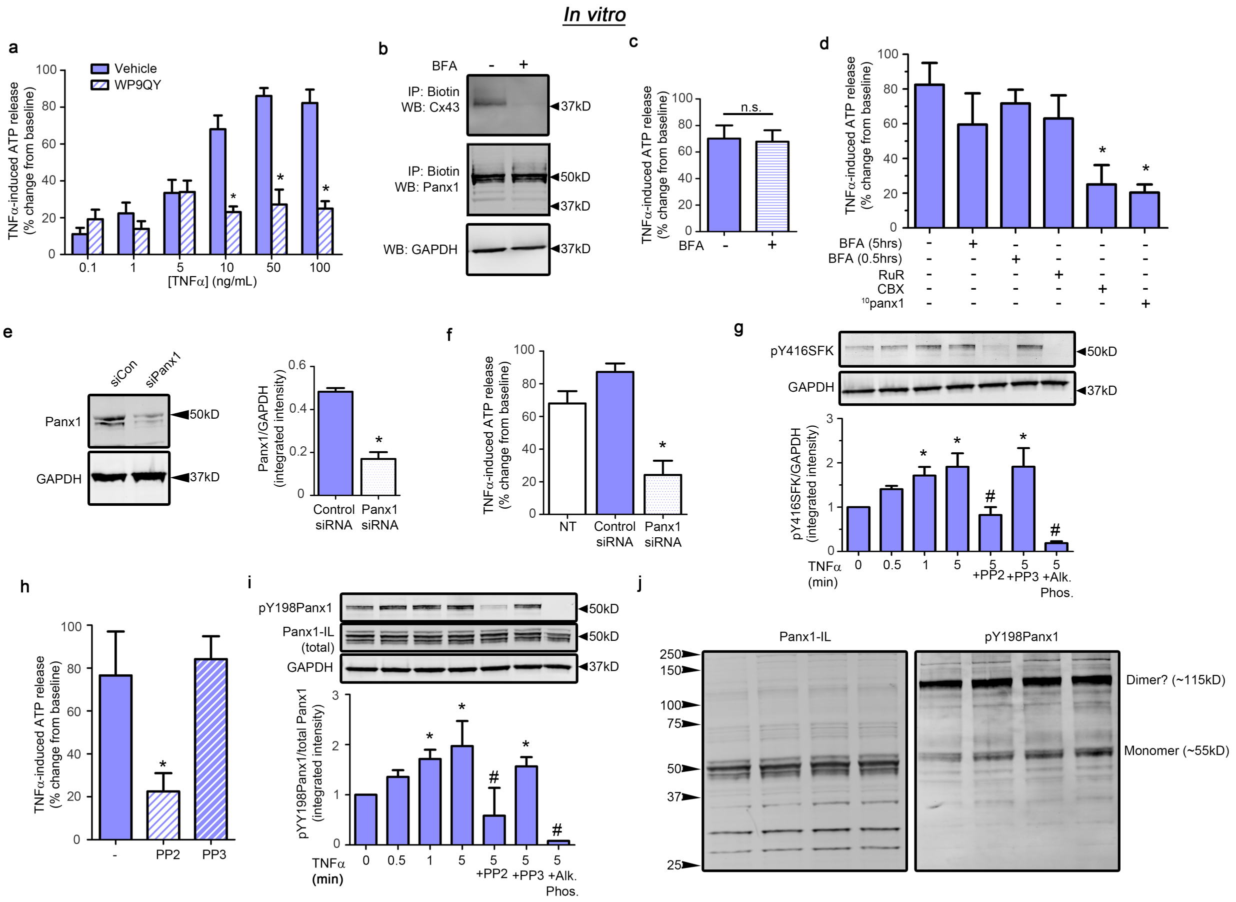

Supplementary Figure 4: HSaVEC release ATP via Panx1 channels in response to TNFα via a SFK-dependent mechanism (a) Dose response of Human Saphenous Vein Endothelial Cells (HSaVEC) to TNFα before and after inhibition of TNFR1 with WP9QY (10 μM). * =p<0.01 as compared to vehicle (n=5). **(b)** Representative Western blot of HSaVEC subjected to treatment with brefeldin A (BFA; 5 μg/mL) for 5 hours and subsequent cell surface biotinylation of membrane proteins. Panx1 and Cx43 plasma membrane localization was assessed. **(c)** ATP release from BFA treated HSaVEC in response to TNFα (10 ng/mL) treatment for 30 minutes. **(d)** Summary data of pharmacological inhibitors assessed for inhibition of TNFα-induced ATP release from HSaVEC. BFA (30min): inhibition of vesicular release; BFA (5hr): inhibition of Cx hemichannels; Ruthenium Red (RuR): antagonist of CALHM1 channels; CBX and

^{10}panx1: Panx1 antagonists. *=$p<0.05$ as compared to HSaVEC treated with TNFα only (n=5). **(e)** Representative Western blot of siRNA knockdown of Panx1 in HSaVEC, and its quantification. *=$p<0.05$ (n=3). **(f)** ATP release from siRNA treated HSaVEC from **(e)** in response to TNFα (10 ng/mL). NT: non-transfected. *=$p<0.05$ (n=4). **(g)** Western blot analysis of SFK activation in HSaVEC in response to TNFα stimulation (10 ng/mL). A phospho-specific antibody against Y416 in SFKs (pY416SFK) was used as an indicator of SFK activation. SFK activation was blocked with the pharmacological antagonist PP2 (10μM) but not by its inactive analog PP3 (10μM). *=$p<0.05$ compared to vehicle control (lane 1) and #=$p<0.01$ compared to 5 min TNFα stimulation (lane 4) (n=3). **(h)** TNFα-induced ATP release from HSaVEC following SFK inhibition with PP2 (10μM). *=$p<0.05$ compared to control and PP3 (n=5). **(i)** Western blot analysis of Panx1 phosphorylation at Y198 in HSaVECs stimulated with TNFα (10 ng/mL). Panx1 phosphorylation was detected using a phospho-specific antibody to Y198 in Panx1 (pY198Panx1). Phospho-signal was normalized to total Panx1 expression using an antibody raised against the non-phosphorylated epitope in the Panx1 intracellular loop (Panx1-IL).*=$p<0.05$ compared to vehicle control (lane 1). #=$p<0.01$ compared to 5 min TNFα stimulation (lane 4) (n=3). **(j)** Representative uncropped Panx1-IL and pY198Panx1 Western blots. The presence of a potential dimer species is shown in the pY198Panx1 blot. All data are presented as mean±SEM (error bars). Statistical analyses were performed using One-way ANOVA.

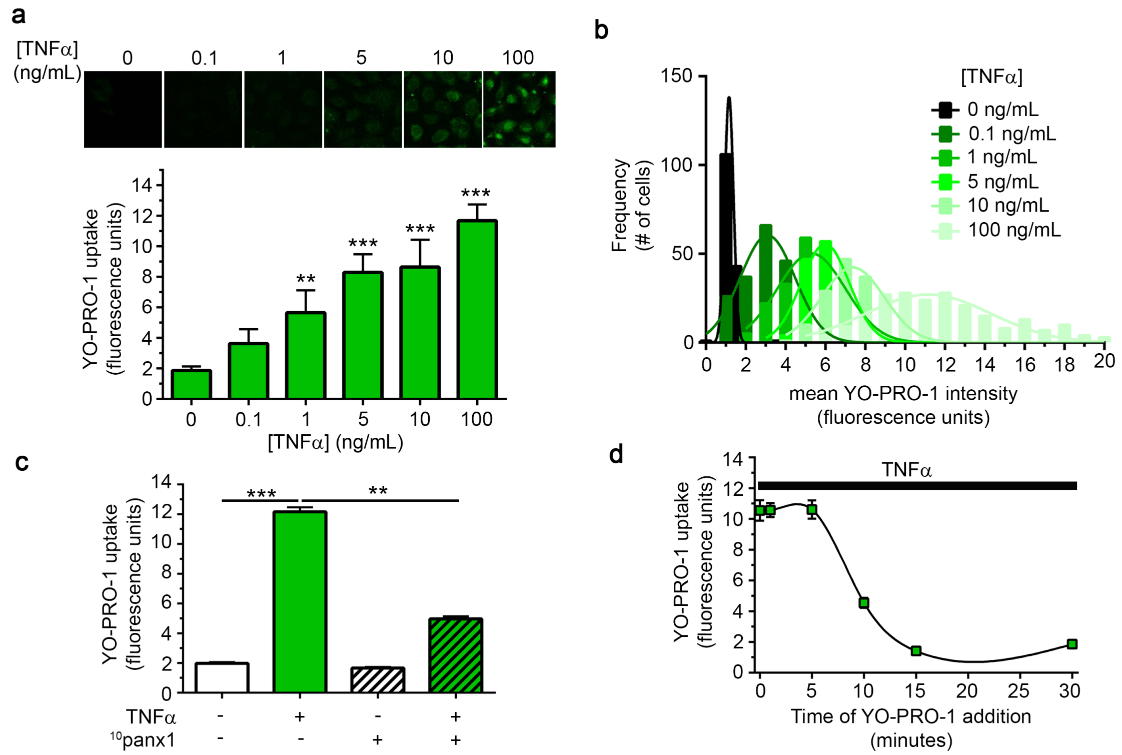

Supplementary Figure 5: TNFα-induced YO-PRO-1 dye uptake by Panx1 channels in HUVEC (a) YO-PRO-1 (1μM) dye uptake by HUVEC stimulated with increasing concentrations of recombinant human TNFα for 30 minutes.**=p<0.01 and ***=p<0.005 compared to non-stimulated controls (n=3)**. (b)** Intensity histogram of YO-PRO-1 fluorescence in HUVEC treated with increasing doses of TNFα. **(c)** Inhibition of YO-PRO-1 uptake by the Panx1 blocker ^{10}panx1 (200μM). HUVEC were treated with TNFα (10ng/mL) for 30 minutes. ***=p<0.005 compared to non-stimulated controls and **=p<0.01 compared to TNFα treated cells in the absence of ^{10}panx1 (n=3). **(d)** Temporal profile of YO-PRO-1 uptake by TNFα stimulated HUVEC. Cells were treated with TNFα (10ng/mL) and YO-PRO-1 was added at various time points following TNFα application. All cells were fixed and imaged 30 minutes after TNFα application. All data are presented as mean±SEM (error bars). Statistical analyses were performed using One-way ANOVA.

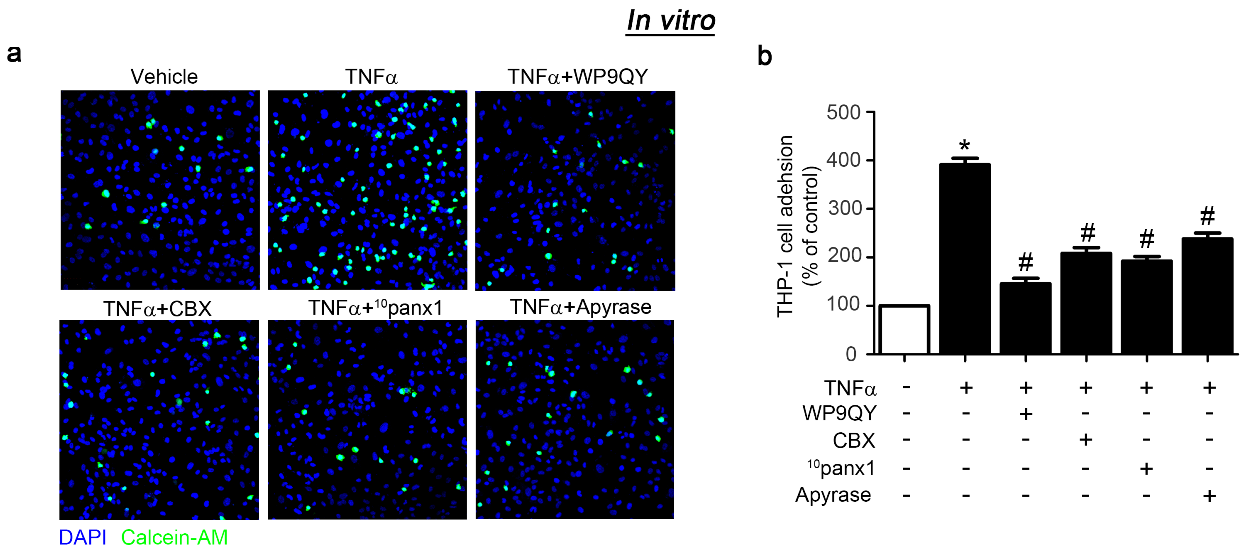

Supplementary Figure 6: Panx1 activation promotes THP-1 monocyte adhesion to cultured ECs (a) THP-1 monocyte adhesion assay on TNFα-stimulated HUVEC. HUVEC nuclei are stained with DAPI in blue and adherent calcein-AM loaded THP-1 monocytes are in green. **(b)** Quantification of TNFα-induced monocyte adhesion in HUVEC treated with WP9QY, CBX, ^{10}panx1 or Apyrase prior to TNFα stimulation. *=p<0.01 compared to vehicle treated cells and #=p<0.01 as compared to TNFα treated cells in the absence of inhibitors by One-way ANOVA (n=5). All data are presented as mean±SEM (error bars).

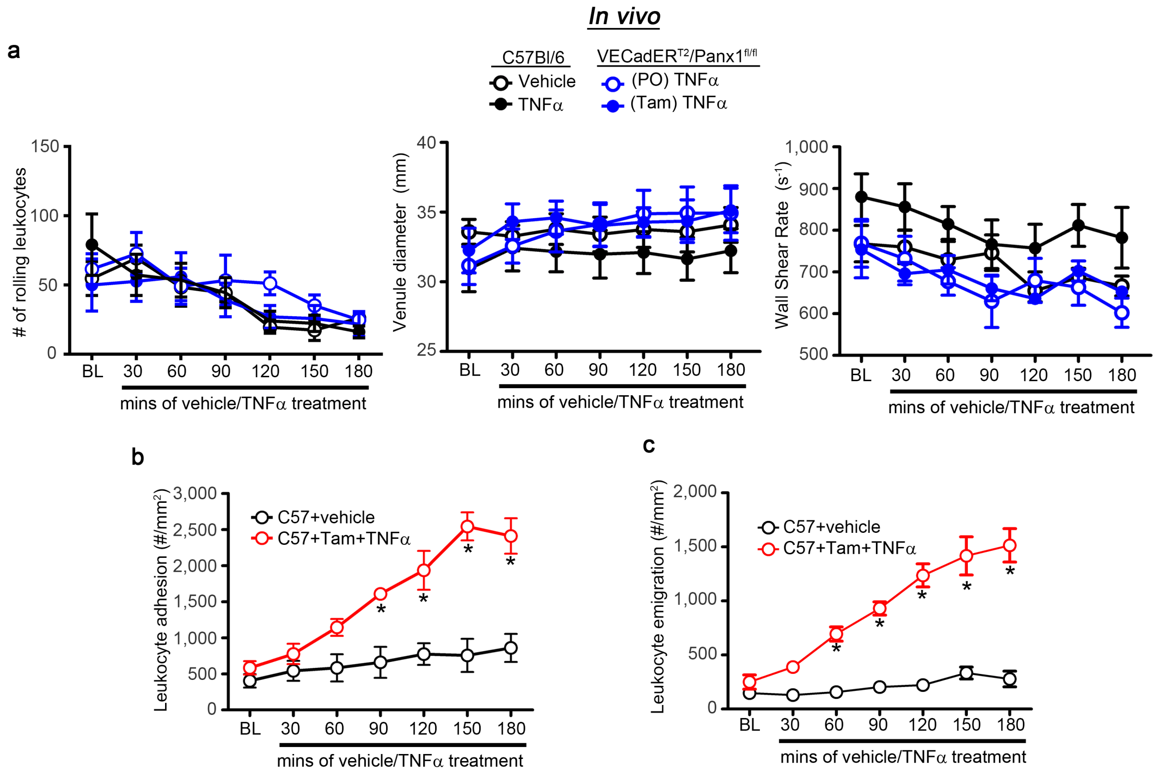

Supplementary Figure 7: Tamoxifen does not affect leukocyte interactions with venous ECs in C57Bl/6J mice (a) Intravital microscopy analysis of the absolute number of rolling leukocytes, post-capillary venule diameter and wall shear rate in C57 and *VECadER*$^{T2+}$*/Panx1*$^{fl/fl}$ mice during TNFα-induced leukocyte adhesion and emigration. **(b-c)** Intravital microscopy analysis of adhesion **(b)** and emigration **(c)** in C57Bl/6J (C57) mice injected with tamoxifen (Tam) for 10 consecutive days. Topical application of TNFα to the exteriorized cremaster muscle resulted in a time dependent increase in leukocyte adhesion to post-capillary venules (#/mm^2 vessel wall) and emigration into the interstitium (#/mm^2 interstitium) in Tam injected C57 mice. *=p<0.01 as compared to C57+Vehicle by One-way ANOVA (n=5). All data are presented as mean±SEM (error bars).

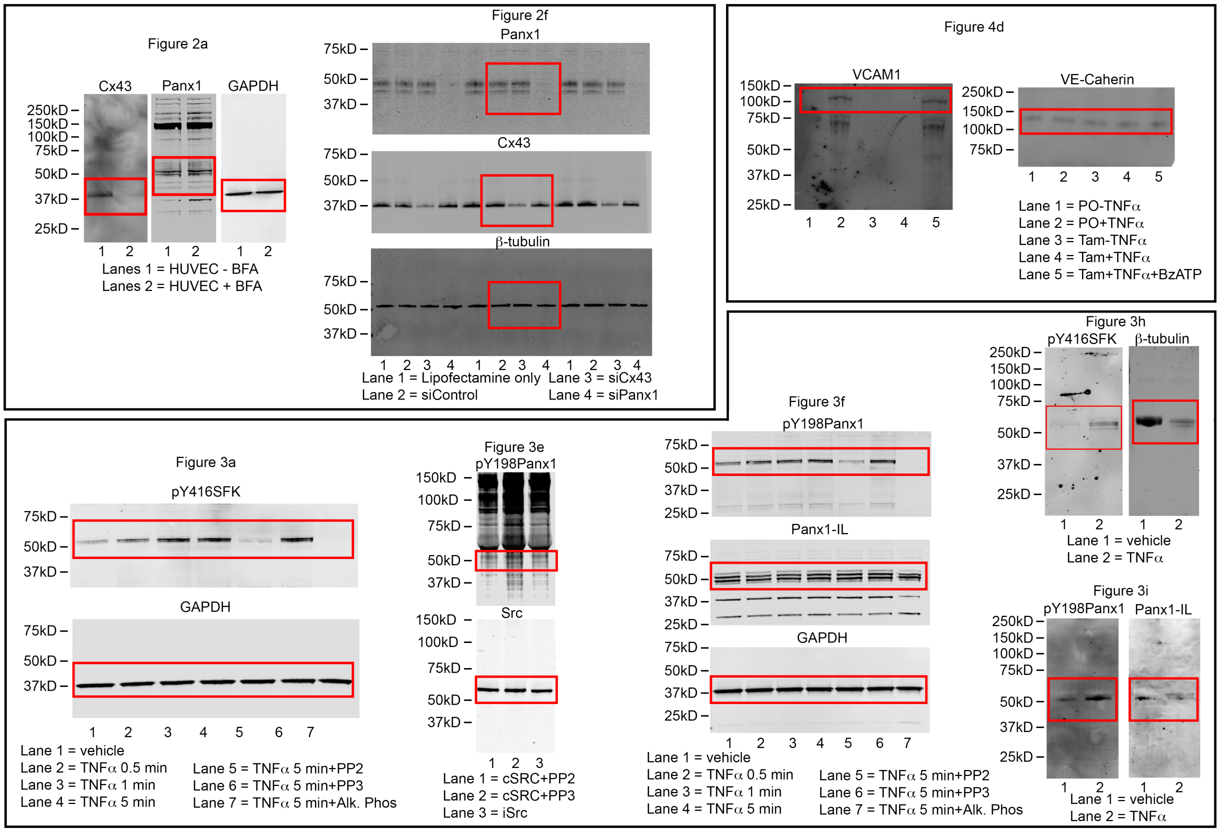

Supplementary Figure 8: Full scans of all western blots presented in cropped form in the main figures. Red rectangles represent areas that were cropped and numbers represent molecular weights in kDa.

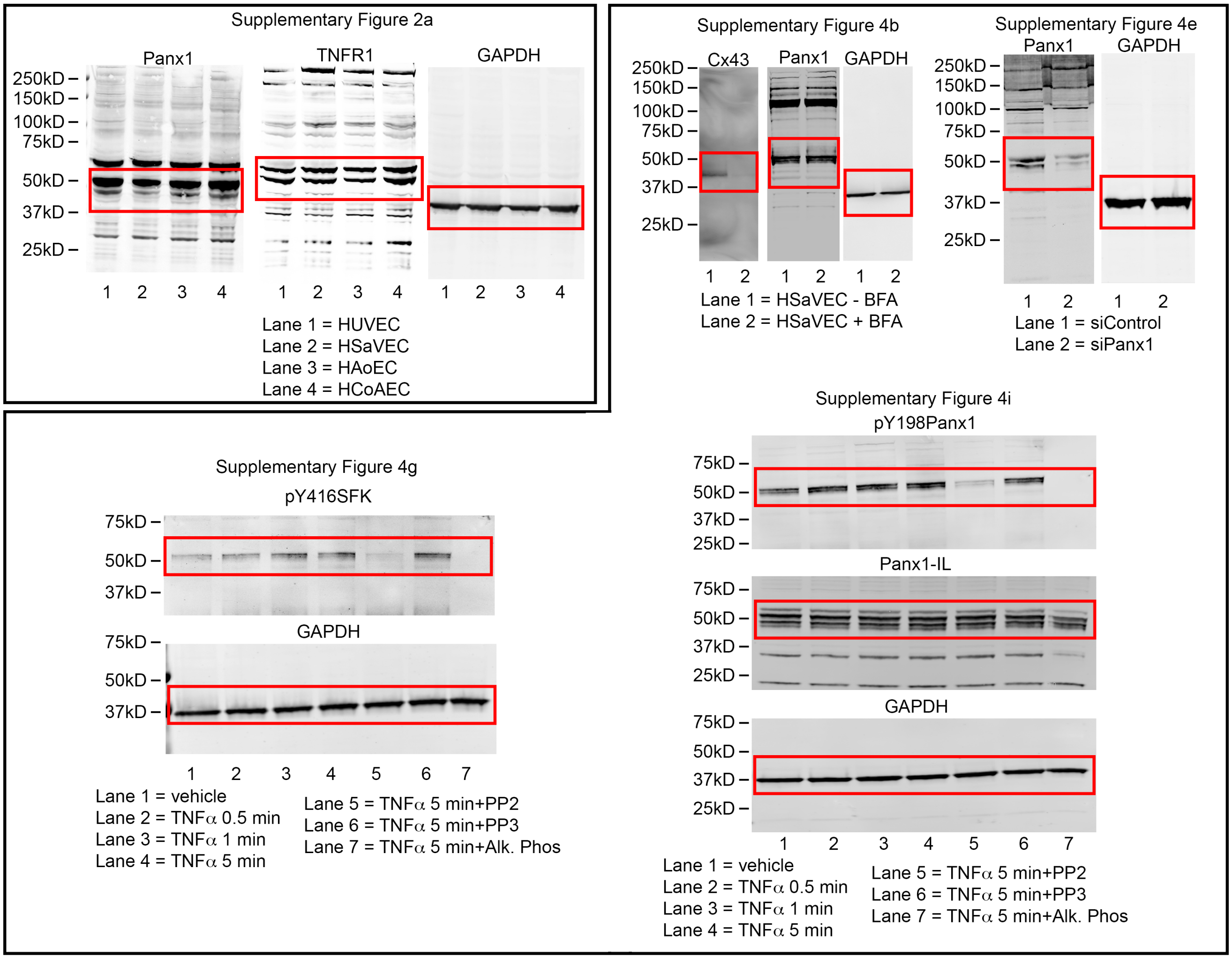

Supplementary Figure 9: Full scans of all western blots presented in cropped form in the supplementary figures. Red rectangles represent areas that were cropped and numbers represent molecular weights in kDa.
